# CD44 regulates Epac1-mediated β-adrenergic-receptor-induced Ca^2+^-handling abnormalities: implication in cardiac arrhythmias

**DOI:** 10.1186/s12929-023-00944-0

**Published:** 2023-07-14

**Authors:** Yi-Hsin Chan, Feng-Chun Tsai, Gwo-Jyh Chang, Ying-Ju Lai, Shang-Hung Chang, Wei-Jan Chen, Yung-Hsin Yeh

**Affiliations:** 1 Cardiovascular Division, Chang-Gung Memorial Hospital, 5 Fu-Hsin Street, Guishan, Taoyuan Taiwan; 2 School of Medicine, College of Medicine, Chang-Gung University, Taoyuan, Taiwan; 3 School of Traditional Chinese Medicine, College of Medicine, Chang-Gung University, Taoyuan, Taiwan; 4 Department of Respiratory Therapy, College of Medicine, Chang-Gung University, Taoyuan, Taiwan; 5 Graduate Institute of Clinical Medical Sciences, College of Medicine, Chang-Gung University, Taoyuan, Taiwan; 6 Division of Cardiovascular Surgery, Kaohsiung Medical University Hospital, Kaohsiung, Taiwan

**Keywords:** CD44, β-Adrenergic receptor, Exchange proteins directly activated by cAMP, Osteopontin, Calcium leak, Cardiac arrhythmia

## Abstract

**Background:**

Sustained, chronic activation of β-adrenergic receptor (β-AR) signaling leads to cardiac arrhythmias, with exchange proteins directly activated by cAMP (Epac1 and Epac2) as key mediators. This study aimed to evaluate whether CD44, a transmembrane receptor mediating various cellular responses, participates in Epac-dependent arrhythmias.

**Methods:**

The heart tissue from CD44 knockout (CD44^−/−^) mice, cultured HL-1 myocytes and the tissue of human ventricle were used for western blot, co-immunoprecipitaiton and confocal studies. Line-scanning confocal imaging was used for the study of cellular Ca^2+^ sparks on myocytes. Optical mapping and intra-cardiac pacing were applied for arrhythmia studies on mice’s hearts.

**Results:**

In mice, isoproterenol, a β-AR agonist, upregulated CD44 and Epac1 and increased the association between CD44 and Epac1. Isoproterenol upregulated the expression of phospho-CaMKII (p-CaMKII), phospho-ryanodine receptor (p-RyR), and phospho-phospholamban (p-PLN) in mice and cultured myocytes; these effects were attenuated in CD44^−/−^ mice compared with wild-type controls. In vitro, isoproterenol, 8-CPT-cAMP (an Epac agonist), and osteopontin (a ligand of CD44) significantly upregulated the expression of p-CaMKII, p-RyR, and p-PLN; this effect was attenuated by CD44 small interfering RNA (siRNA). In myocytes, resting Ca^2+^ sparks were induced by isoproterenol and overexpressed CD44, which were prevented by inhibiting CD44. Ex vivo optical mapping and in vivo intra-cardiac pacing studies showed isoproterenol-induced triggered events and arrhythmias in ventricles were prevented in CD44^−/−^ mice. The inducibility of ventricular arrhythmias (VAs) was attenuated in CD44^−/−^ HF mice compared with wild-type HF controls. In patients, CD44 were upregulated, and the association between CD44 and Epac1 were increased in ventricles with reduced contractility.

**Conclusion:**

CD44 regulates β-AR- and Epac1-mediated Ca^2+^-handling abnormalities and VAs. Inhibition of CD44 is effective in reducing VAs in HF, which is potentially a novel therapeutic target for preventing the arrhythmias and sudden cardiac death in patients with diseased hearts.

**Supplementary Information:**

The online version contains supplementary material available at 10.1186/s12929-023-00944-0.

## Introduction

The human heart expresses β-adrenergic receptors (β-AR), which are essential to increase heart rate and cardiac contractility upon physiological stimulation. The heart has both β1- and β2-AR and β1-AR is the predominant type. However, chronic sustained activation of β-adrenergic receptors (β-ARs) is detrimental and may induce events of life-threatening ventricular tachycardia/fibrillation, which is the leading cause of sudden cardiac death in patients with diseased heart and heart failure (HF) [1, 2].

Sustained β-AR activation provokes ventricular arrhythmias (VAs) as a result of abnormal Ca^2+^ leakage from the sarcoplasmic reticulum (SR). Activation of β-AR converts adenosine triphosphate (ATP) into cyclic adenosine monophosphate (cAMP), through which β-AR transmits signals to two target proteins: protein kinase A (PKA) and exchange proteins directly activated by cAMP (Epac) [3]. Both PKA and Epac activate Ca^2+^-handling proteins, including ryanodine receptors (RyR2) and phospholamban (PLN), leading to abnormal Ca^2+^ leakage from the SR and thus triggering arrhythmic events [3]. Hyperphosphorylation of RyR2 and PLN leads to Ca^2+^ leakage from the SR during sustained β-AR activation, which is mediated by either activation of PKA or Ca^2+^/calmodulin-dependent protein kinase II (CaMKII).

Epac mediates the cAMP-dependent β-AR/CaMKII axis in the heart [4]. There are two isoforms of Epac: Epac1 and Epac2. Epac1 is ubiquitous with high expression levels in the heart and kidneys, while Epac2 has a more restricted expression. They both are present in the heart and have been shown to mediate cardiac arrhythmias [5, 6]. Targeting Epac is a proven strategy in preventing cardiac arrhythmias [7].

Osteopontin is a glycoprotein synthesized by various tissues and cells including cardiomyocytes, whose level is elevated in diseased heart [8, 9]. Increased osteopontin induces adverse cardiac remodeling, such as the fibrosis of the heart tissue [10]. CD44, a transmembrane protein mediating various cell responses, is a receptor of osteopontin [11, 12]. Recently, our studies showed that CD44 is involved in atrial fibrillation (AF) pathophysiology related to Ca^2+^-handling proteins and ion channel remodeling [13, 14]. Since β-AR activation plays an important role in cardiac arrhythmias, we evaluated, in this study, whether CD44 is involved in β-AR-induced arrhythmias. We show that osteopontin/CD44 signaling is involved in β-AR-mediated activation of Ca^2+^-handling proteins and Ca^2+^ leakage from SR by interacting with Epac1.

## Methods

See Additional file 1: Methods.

## Results

### CD44 is involved in β-AR activation on Ca^2+^-handling proteins

**Fig. 1:**
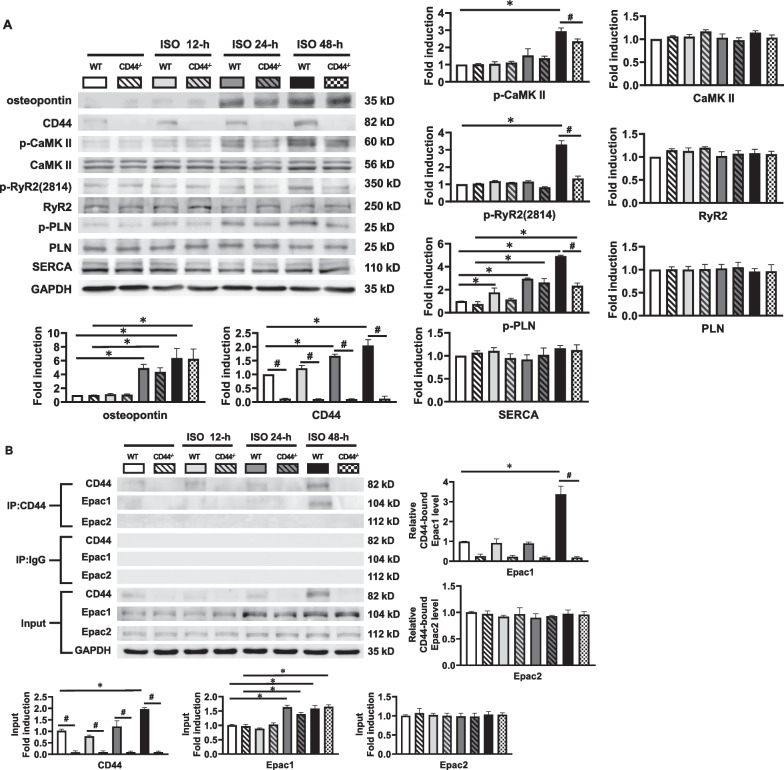

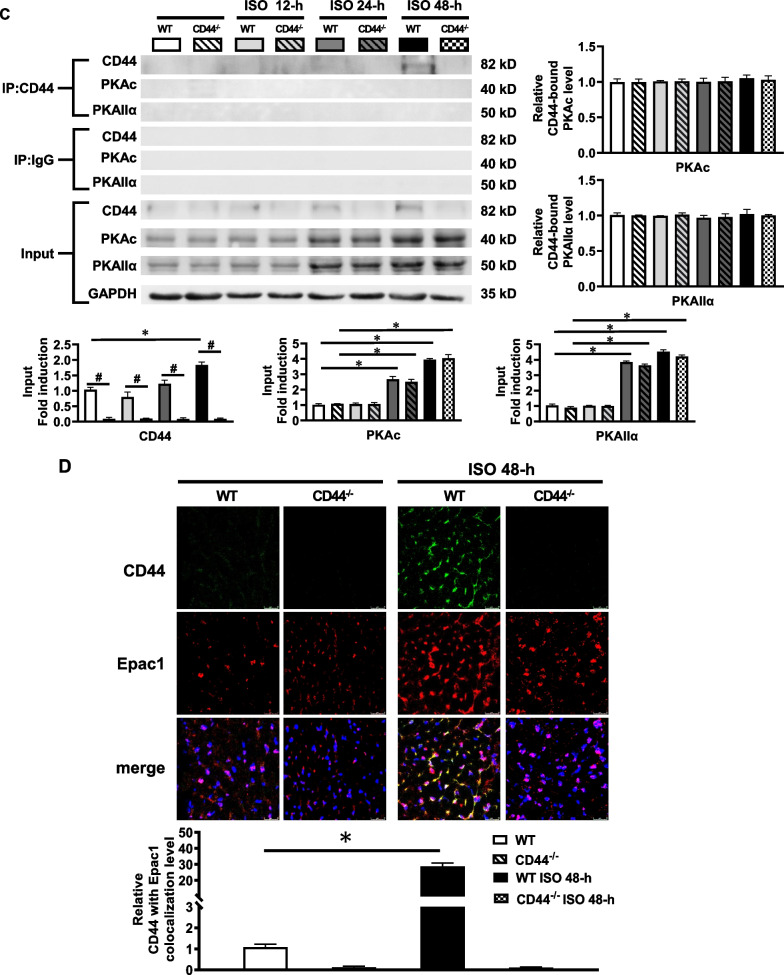
Expression of osteopontin, CD44, CaMKII and Ca^2+^-handling proteins in β-AR-activated CD44^−/−^ mice at different time points during treatment with β-AR agonist. **A** Representative examples and mean ± SE analysis western blot for osteopontin, CD44, ox-CaMKII, (p-)CaMKII, (p-)RyR2, SERCA, NCX and (p-)PLN. The relative expression of each protein was quantified to GAPDH by densitometry and normalized to the control. *N* = 4 for each group. **B** Representative examples and mean ± SE analysis for co-immunoprecipitation of CD44 and control IgG with Epac1 and Epac2. **C** Representative examples and mean ± SE analysis for co-immunoprecipitation of CD44 and PKAc and PKAIIα. In **B** and **C**, the pictures are representation of blots from 3 independent experiments for each. the mean ± SE analysis was for precipitated protein-bound CD44 and Epac1 to precipitated protein ratio and cell input, both of which were quantified to GADPH and normalized to the control (WT without ISO) level, which was set at 1.0. *N* = 3 for each group. For **A**–**C** *P < 0.05 versus control (WT and CD44^−/−^ mice without ISO) by one-way ANOVA with Bonferroni’s post hoc test. ^#^*P* < 0.05 between groups by unpaired Student’s t-test. **D** Representative co-focal images of CD44 (upper, green color), Epac1 (middle, red color) and co-localization of both (lower, yellow color) and mean ± SE analysis of co-localization in WT and CD44^−/−^ mice heart after 48-h ISO. The relative fluorescence of co-localization was normalized to WT mice as 1.0. *N* = 4 per group. For **D**, *P < 0.05 versus control by one-way ANOVA with Bonferroni’s post hoc test. WT = wild-type control, CD44^−/−^ = CD44 knock-out mice, ISO = isoproterenol at 30 mg/kg per day subcutaneously

Sustained adrenergic stimulation causes hyperactivation of Ca^2+^-handling proteins and contributes to detrimental cardiac arrhythmias [1]. To investigate the significance of CD44 induced by sustained β-AR activation, we first evaluated the expression of CD44 and Ca^2+^-handling proteins in mice heart treated with daily high-dose isoproterenol at different time points. Both the wild-type control (WT) and CD44^−/−^ mice were given daily boluses of isoproterenol at 30 mg/kg subcutaneously. The heart was harvested without isoproterenol, and at 12 h, 24 h and 48 h after the first bolus of isoproterenol. As shown in Fig. 1A, CD44 was upregulated in WT mice at 48 h after treatment with isoproterenol, and the expression level of osteopontin, which is a ligand of CD44, was significantly elevated in WT and CD44^−/−^ mice.

We also evaluated the effect of a single bolus of high-dose isoproterenol at a short interval. The expression of p-RyR2 (2814) and p-PLN was evaluated before and at 1, 3, 6, and 12 h after isoproterenol in both WT and CD44−/− mice. As shown in Additional file 2: Figure S1, isoproterenol caused a significant increase in p-RyR2 and p-PLN at 1, 3, and 6 h in both WT and CD44-/- mice. The expression of p-RyR2 recovered to baseline, but p-PLN was still upregulated at 12 h. Taken together, the results indicated that a single bolus of high-dose isoproterenol caused a quick but non-sustained upregulation of p-RyR2 and a sustained increase of p-PLN, whereas repeated boluses caused a sustained increase of both at 48 h in WT mice, which was significantly reduced in CD44^−/−^ mice.

Since the results suggested 48-h isoproterenol upregulates CD44, we further evaluated if upregulated CD44 is involved in hyperactivation of Ca^2+^-handling proteins induced by sustained β-AR-activation. CaMKII is the target of Epac. The expression level of activated CaMKII (p-CaMKIIδ) was significantly elevated in isoproterenol-treated WT mice at 48 h after isoproterenol, which was abolished in CD44^−/−^ mice. β-AR activation can induce phosphorylation of RyR2 Ser-2814 (the site phosphorylated by CaMKII) [15] and PLN through PKA and CaMKII. CaMKII is the target of Epac1 and Epac2. The expression levels of CaMKII, RyR2, SERCA, and NCX were similar between the isoproterenol-treated CD44^−/−^ and WT mice.

Both PKA and Epacs are the targets of β-AR. We further evaluated if β-AR activation provokes the association between CD44 and Epacs or PKA. Co-immunoprecipitation was performed to evaluate the association between CD44 and Epac1/Epac2 and PKA subunits (PKAc and PKAIIα) in isoproterenol-treated CD44^−/−^ mice and wild-type controls. The lysates of ventricle tissue samples were immunoprecipitated using anti-CD44 antibodies. The immunocomplexes were resolved and reblotted with anti-Epac 1/2, anti-PKAc, anti-PKAIIα, or anti-CD44 antibodies. As shown in Fig. 1B, the expression levels of CD44 and Epac1 were significantly increased in WT but not in CD44^−/−^ mice treated with isoproterenol. Isoproterenol did not change the expression level of Epac2 in WT or CD44^−/−^ mice. The association between CD44 and Epac1 was thus trivial in CD44^−/−^ mice and WT. In WT mice, β-AR activation by isoproterenol induced increased expression of CD44 and Epac1, as well as increased association between CD44 and Epac1. The analysis showed the ratio of CD44-associated Epac1 to CD44 was also increased. The interaction between CD44 and Epac2 was trivial and unchanged in WT treated with isoproterenol. Reverse co-immunoprecipitation was performed using anti-Epac1 antibody to precipitate the lysates, which were then reblotted with anti-CD44 antibody. It confirmed that the association between Epac1 and CD44 was increased by isoproterenol. (Additional file 3: Figure S2) Fig. 1C shows that the expression level of PKAc was significantly elevated by isoproterenol treatment in WT mice. There was little interaction between CD44 and PKAc or PKAIIα in WT and CD44^−/−^ with or without β-AR activation by isoproterenol. We also evaluated if CD44 and Epac1 co-localize in the heart after β-AR activation by confocal immunofluorescence, as previously described [16]. Figure 1D shows that compared with CD44^−/−^ mice, the expression of CD44 and Epac1 and the colocalization between the two were significantly increased in WT mice after treatment with 48-h isoproterenol. Taken together, the results suggested that: (1) CD44 regulates β-AR-mediated activation of CaMKII and Ca^2+^-handling proteins, including RyR2 and PLN, via Epac1 after a prolonged (48 h) time of β-AR activation, and (2) β-AR activation increases the association between CD44 and Epac1. In contrast, the results showed a lack of interaction between CD44 and PKA subunits and that the expression levels of PKA subunits increased similarly in both β-AR-activated CD44^−/−^ mice and WT; the results suggest that CD44 is likely not involved in PKA-mediated signal transduction.

### β-AR activation and osteopontin activate Ca^2+^-handling proteins via CD44 and Epac1

**Fig. 2:**
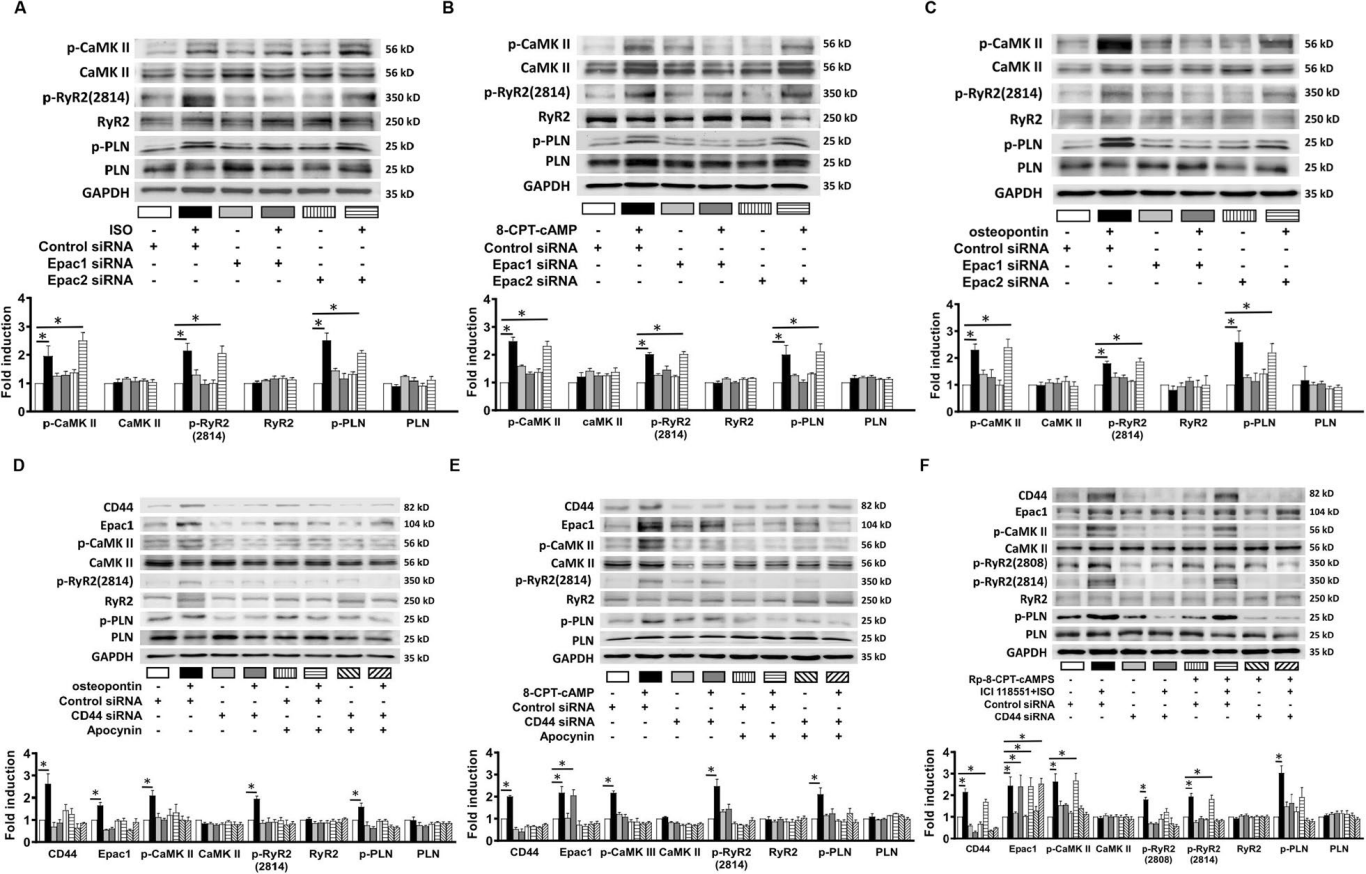
Crosstalk between β-AR/Epac and osteopontin/CD44 signaling on Ca^2+^-handling proteins. **A–C** Representative examples and mean ± SE analysis western blot of (p-)CaMKII, (p-)RyR2 and (p-)PLN in HL-1 myocytes treated with **(A)** isoproterenol **(B)** 8-CPT-cAMP **(C)** osteopontin and with or without Epac1 or Epac2 siRNA. **D** and **E** Representative examples and mean ± SE analysis western blot of CD44, Epac1, (p-)CaMKII, (p-)RyR2 and (p-)PLN in HL-1 myocytes treated with **(D)** osteopontin or **(E)** 8-CPT-cAMP and with or without CD44 siRNAs or apocynin. **F** Representative examples and mean ± SE analysis western blot of CD44, Epac1, (p-)CaMKII, (p-)RyR2 and (p-)PLN in HL-1 myocytes treated with isoproterenol + ICI-118,551 with or without CD44 siRNAs or Rp-8-CPT-cAMPS. The relative expression of each protein was quantified to GAPDH by densitometry and normalized to the control. For all **A**–**F**, *n* = 4 per group. *P < 0.05 versus control by one-way ANOVA with Bonferroni’s post hoc test. ISO = isoproterenol

Osteopontin is the ligand of CD44 and has previously been shown to be increased in the diseased heart [9]. Therefore, we wanted to evaluate whether osteopontin may contribute to β1-AR-induced activation of CaMKII and Ca^2+^-handling proteins via the interaction between CD44 and Epac1. 8-CPT-cAMP is cell-permeable cAMP analogue used to activate Epac. Cultured HL-1 myocytes were treated with osteopontin (100 ng/mL), isoproterenol (10 µmol/L) or 8-CPT-cAMP (100 µmol/L) for 24 h. Epac1 or Epac2 were blocked by respective targeted siRNAs. The efficacy and specificity of Epac1, Epac2, and CD44 siRNAs are shown in Additional file 4: Figure S3. In Additional file 5: Figure S4, it shows that osteopontin, β-AR activation by isoproterenol, and Epac activation by 8-CPT-cAMP significantly upregulated the expression of Epac1, which was substantially suppressed by Epac1 siRNA. In contrast, the expression level of Epac2 was not altered by osteopontin, isoproterenol, or 8-CPT-cAMP. The results suggest that osteopontin and β-AR activation may jointly target Epac1 rather than Epac2. Figure 2A shows that β-AR activation by isoproterenol significantly upregulated the expression of p-CaMKII, p-RyR2, and p-PLN, which was prevented by Epac1 siRNA. In contrast, Epac2 siRNA was not effective in preventing isoproterenol-induced upregulation of these elements. Similarly, Fig. 2B and C shows that Epac activation by 8-CPT-cAMP and osteopontin significantly induced the expression of p-CaMKII, p-RyR2, and p-PLN, which was prevented by Epac1 siRNA, but not by Epac2 siRNA. The results suggest that osteopontin, β-AR and Epac activation may jointly contribute to the activation of CaMKII, RyR2 and PLN through Epac1 rather than Epac2.

CD44 is the receptor for osteopontin, therefore, we further evaluated whether CD44 is involved in osteopontin-, β-AR- and Epac1-mediated activation of CaMKII, RyR2 and PLN. CD44 siRNA was used to eliminate CD44. The results indicated that the upregulated expression of CD44, Epac1, p-CaMKII, p-RyR2, and p-PLN by osteopontin or 8-CPT-cAMP, was substantially suppressed by CD44 siRNA (Fig. 2D, E).

We would like to evaluate if elimination of CD44 or inhibition of PKA is effective in reducing selective β1-adrenergic activation of Ca^2+^ handling proteins. HL-1 myocytes were treated with isoproterenol and ICI-118,551 at 10 µmol/L, which is a selective β2-AR antagonist, for 24 h. Figure 2F showed that elimination of CD44 by CD44 siRNA significantly reduced the upregulation of p-CaMKII, p-RyR2 Ser2808 (the target site of CaMKII and PKA) [17], p-RyR2 Ser2814 (the target site of CaMKII) [15], and p-PLN by isoproterenol in the presence of ICI-118,551. PKA is known to participate in β1-AR activation of Ca^2+^ handling proteins in parallel to CaMKII^1^. To evaluate the role of PKA in β1-AR activation, we evaluated the effects of Rp-8-CPT-cAMPS, which is a specific PKA inhibitor, on Ca^2+^-handling proteins in HL-1 myocytes treated with isoproterenol in the presence of ICI-118,551. Figure 2F showed that inhibition of PKA by Rp-8-CPT-cAMPS (at 10 µmol/L) did not prevent the upregulation of p-RyR2 Ser2808, p-RyR2 Ser2814, and p-PLN induced by selective β1-AR activation. Taken together, the results suggest that inhibition of CD44 can prevent the activation of Ca^2+^-handling proteins induced by β-AR activation through inhibition of CaMKII. Inhibition of PKA alone may not fully prevent selective β1-AR activation of Ca^2+^-handing proteins.

Overall, the results suggest that osteopontin/CD44 and β-AR/Epac1 signaling may interact to activate CaMKII and downstream RyR2 and PLN.

Since β-AR activation may induce oxidative stress and CD44 is known to regulate NADPH oxidase (NOX) activity, we wanted to evaluate whether inhibition of oxidative stress would affect Epac activation of Ca^2+^-handling proteins. Apocynin (0.5 mmol/L) treatment for 24 h was subsequently applied to evaluate this. Figure 2E showed that inhibition of NOX by apocynin prevented the activation of CaMKIIδ and the downstream RyR2 and PLN induced by Epac activation. Consistent with our previous study, the results show that oxidative stress is also essential in mediating the activation of Ca^2+^-handling proteins.

### Interaction between CD44 and Epac1

**Fig. 3:**
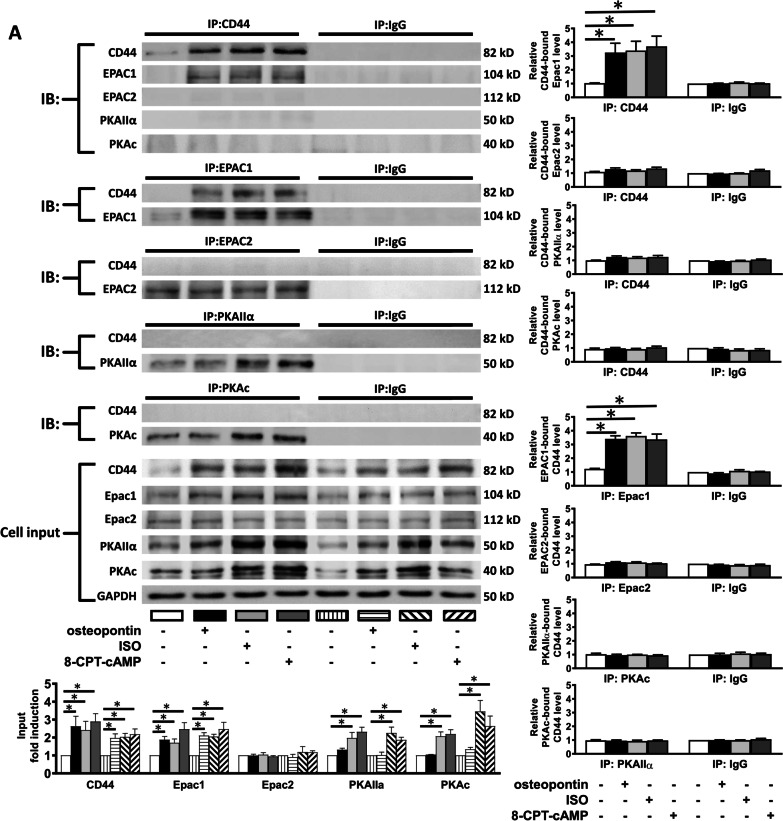

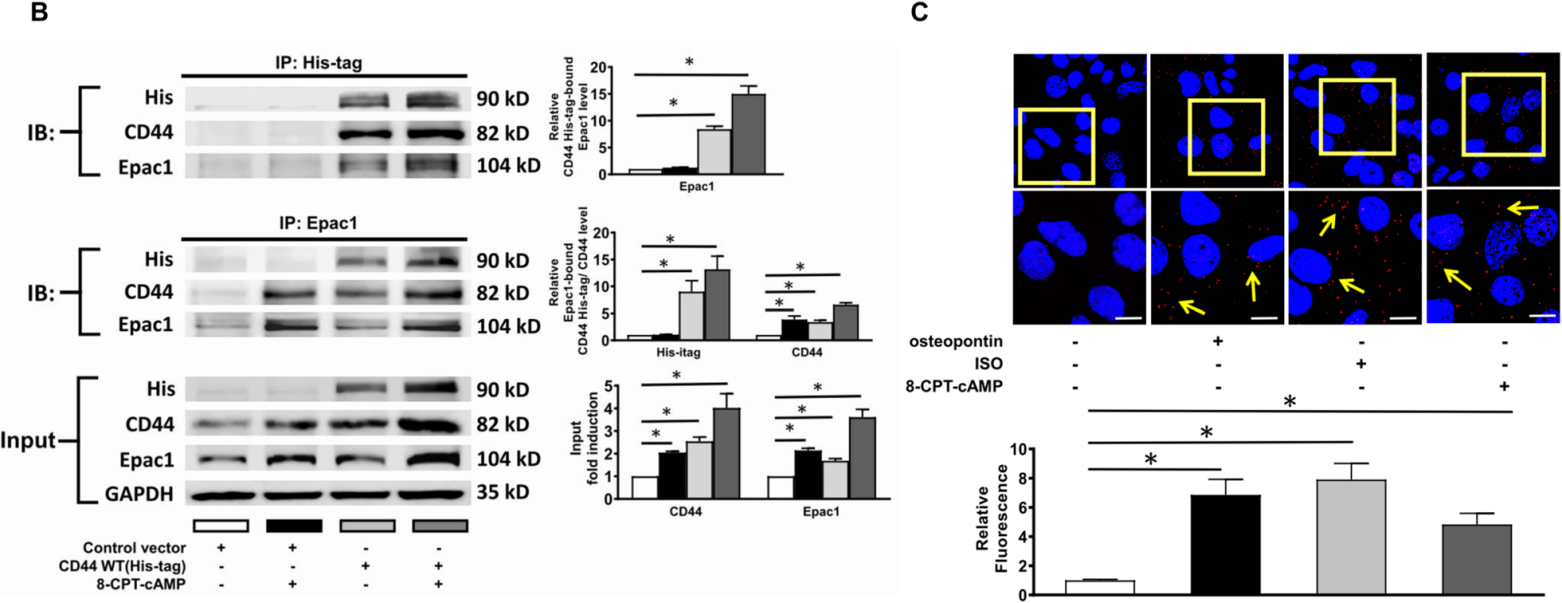
Association between CD44 and Epac1. **A** Representative examples and mean ± SE analysis for co-immunoprecipitation, reverse co-immunoprecipitation and cell input of CD44 and Epac1, Epac2, PAKIIα and PKAc in HL-1 myocytes treated with osteopontin, isoproterenol or 8-CPT-cAMP. **B** Representative examples and mean ± SE analysis for co-immunoprecipitation and cell input of His-tagged CD44 and Epac1 in HL-1 myocytes treated with 8-CPT-cAMP. In **A** and **B** the mean ± SE analysis was for precipitated protein-bound Epac1, Epac2, PAKIIα or PKAc or CD44 to precipitated protein ratio and cell input, both of which were quantified to GADPH and normalized to the control. ISO = isoproterenol. The picture is a representation of blots from 3 independent experiments. In **A** and **B**, *n* = 3 per group. **C** Representative examples and mean ± SE confocal images of proximity ligation assay in HL-1 myocytes. The red color (arrows) indicated the linkage between CD44 and Epac1. The blue color indicated nuclei. The relative fluorescence was normalized to control as 1.0. *n* = 4 images per group. For **A**–**C**, *P < 0.05 versus control by one-way ANOVA with Bonferroni’s post hoc test. ISO = isoproterenol

We performed co-immunoprecipitation to evaluate if osteopontin and activation of β-AR or Epac1 would facilitate the interaction among CD44, Epac, and PKA subunits. HL-1 myocytes were treated with osteopontin (100 ng/mL), isoproterenol (10 µmol/L) or 8-CPT-cAMP (100 µmol/L) for 24 h. The lysates of HL-1 myocytes were obtained and analyzed as input. Co-immunoprecipitation was performed using anti-CD44 antibodies to immunoprecipitate the lysates. Anti-IgG antibody was used as negative control. The immunocomplexes were resolved and reblotted with anti-Epac1/2, anti-PKAIIα, anti-PKAc, and anti-CD44 antibodies. Reverse co-immunoprecipitation used anti-Epac1/Epac2, anti-PKAIIα and anti-PKAc antibodies to immunoprecipitate the lysates. Figure 3A shows that the expression of CD44 and Epac1 as well as the association between CD44 and Epac1 was increased by β-AR activation induced by isoproterenol, Epac activation by 8-CPT-cAMP and osteopontin. The analysis shows the ratio of CD44-associated with Epac1 to CD44 was increased by β-AR and Epac activation, and osteopontin. In contrast, the interaction between CD44 and Epac2, PKAc or PKAIIα was trivial and not changed by any of the treatment conditions investigated.

To evaluate the association between CD44 and Epac1 more surely, wild type His-tag CD44 cDNA-containing plasmids were transfected in HL-1 myocytes. In Fig. 3B, co-immunoprecipitation showed 8-CPT-cAMP increased the expression of CD44 and Epac1 as well as the association between His-tagged CD44 and Epac1. The analysis showed the ratio of His-tagged CD44-associated Epac1 to His-tagged CD44 and the ratio of Epac1-associated His-tagged CD44 and CD44 to Epac1 were also increased by 8-CPT-cAMP. In Fig. 3C, proximity ligation assay was applied to evaluate the interaction between proteins in situ. It showed that the association between CD44 and Epac1 was significantly increased (which is indicated by red color) by osteopontin, isoproterenol, and 8-CPT-cAMP in HL-1 myocytes. Confocal immunofluorescence also confirmed the co-localization between CD44 and Epac1 induced by isoproterenol (1 µmol/L for 24 h). (Additional file 6: Figure S5) Both proximity ligation assay at z-stacking mode and colocalization of CD44 and Epac1 by immunofluorescence suggest that the association between CD44 and Epac1 was located in the cytoplasm and perinuclear regions rather than along the cell membrane.

Overall, the results suggest that osteopontin/CD44 and β1-AR/Epac1 activation may jointly contribute to the activation of CaMKII, RyR2, and PLN through direct interaction between CD44 and Epac1.

### CD44 mediates β-adrenergic-induced SR Ca^2+^ leak

**Fig. 4:**
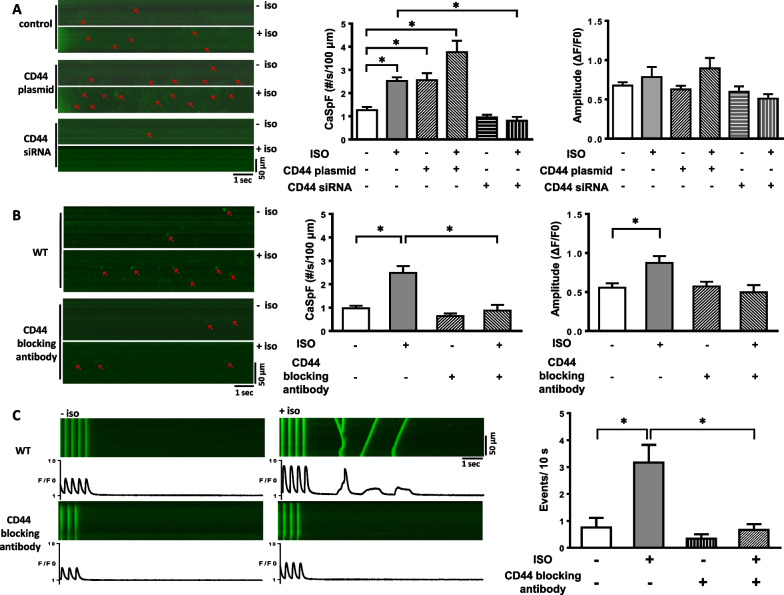
Effect of CD44 and β-AR signaling on the Ca^2+^ sparks and Ca^2+^ waves in myocytes. Representative examples and mean ± SE analysis for the frequency and amplitude of **A** Ca^2+^ sparks (arrow) in HL-1 myocytes transfected with CD44 siRNA or wild-type CD44 cDNA -containing plasmids. *n* = 17–28 cells for each group. **B** Ca^2+^ sparks (arrow) in ventricular myocytes from wild-type mice (WT) treated with CD44 blocking antibody and **C** Ca^2+^ waves in ventricular myocytes from WT after 3-Hz electrical stimulation. In **B** and **C**, *n* = 4–20 cells for each group. In **A**–**C**, all were obtained before and after isoproterenol (1 μmol/L) infusion with and without treatment with CD44 blocking antibody. *P < 0.05 versus control and between ISO and ISO + CD44 siRNA or CD44 blocking antibody by one-way ANOVA with Bonferroni’s post hoc test. CaSpF = Ca^2+^ spark frequency, ISO = isoproterenol, WT = wild-type mice

Next, we sought to determine whether CD44 is involved in β-adrenergic-induced SR Ca^2+^ leakage in cardiomyocytes. CD44 plasmid-containing vectors or CD44 silencing with its respective siRNA were transfected into HL-1 myocytes, and after 24 h the cells were used for the experiments. Figure 4A shows that the Ca^2+^ spark frequency (CaSpF) was significantly increased in HL-1 myocytes acutely treated with isoproterenol at 1 µmol/L. CaSpF was further increased when CD44 was more highly expressed by the CD44 plasmid-containing vectors. In contrast, CaSpF was substantially suppressed when CD44 was reduced by CD44 siRNA. Similarly, Fig. 4B shows that isoproterenol significantly increased CaSpF in isolated ventricular myocytes from the WT mice. The effect of isoproterenol was abolished when CD44 was inhibited by anti-CD44 blocking antibodies.

To how abnormal SR Ca^2+^ release events might cause arrhythmias, isolated WT ventricular myocytes were given a high-frequency pacing train (3 Hz for 30 s) followed by a pause. Figure 4C shows that isoproterenol resulted in an increased frequency of spontaneous Ca^2+^ waves in WT myocytes, which was significantly reduced when the myocytes were treated with anti-CD44 blocking antibodies.

We used the RyR inhibitors flecanide, tocanide, and tetracaine in isolated cardiomyocytes from WT mice to determine if the calcium waves elicited by isoproterenol were RyR-dependent [18, 19]. The isolated myocytes were incubated with flecanide (10 μM) for 20 min, tocanide (50 μM), or tetracaine (50 μM) for 10 min before 3-Hz pacing and isoproterenol. As shown in Additional file 7: Figure S6, the calcium waves induced by isoproproterenol in WT myocytes were virtually eliminated by flecanide, tocanide and tetracaine, which suggests the induced calcium waves were RyR-dependent.

The results thus demonstrate that CD44 is a key determinant of aberrant Ca^2+^ homeostasis in cardiomyocytes in response to β-AR/Epac activation.

### CD44 mediates β1-adrenergic-induced ventricular arrhythmia

**Fig. 5:**
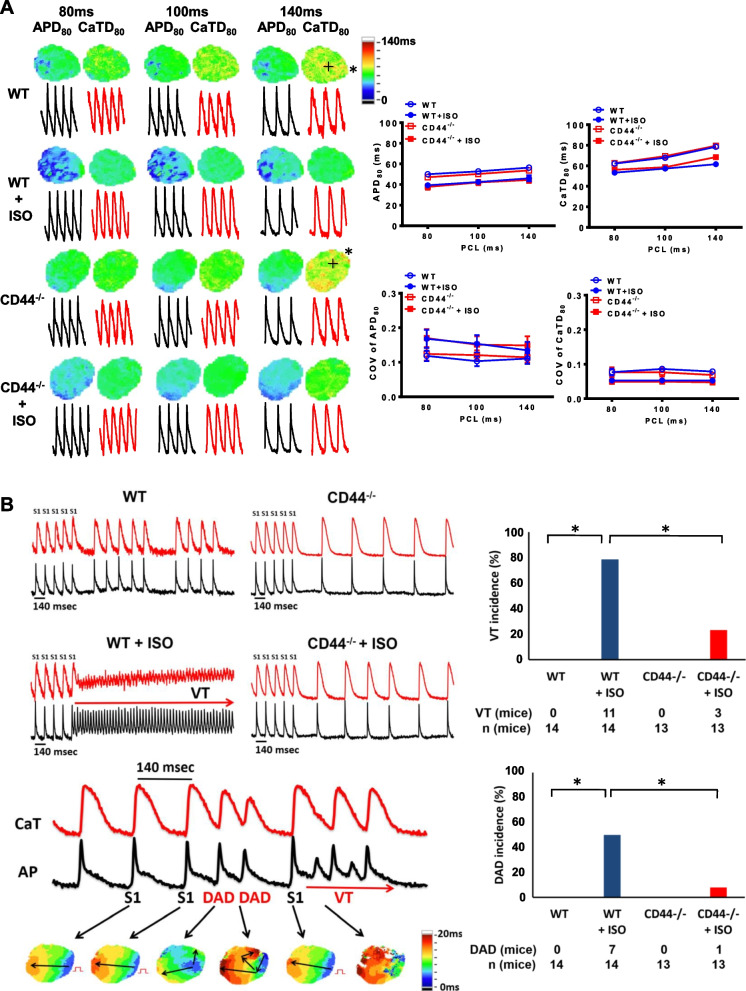
Optical mapping in CD44^−/−^ mice. **A** Representative examples (left panel) and mean ± SE analysis (right panel) of optical action potential duration (APD) and calcium transient duration (CaTD) maps of wild-type (WT) and CD44^−/−^ mice at different pacing cycle length (PCLs). **B** Representative examples (left panel) and mean ± SE analysis (right panel) of ventricular tachyarrhythmia (VT) and delayed after depolarizations (DADs) in WT and CD44^−/−^ mice. In **A** and **B**, both were before and after isoproterenol (10 nmol/L) infusion. *N* = 13–14 mice for each group. In **B,** *P < 0.05 between groups by Fisher’s exact test. CD44^−/−^ = CD44 knock-out mice, ISO = isoproterenol, WT = wild-type mice, APD_90_ = APD at 80% repolarization, CaTD_80_ = CaTD at 80 repolarization, COV = coefficient of variance, PCL = pacing cycle length, VT = ventricular tachyarrhythmia, DAD = delayed afterdepolarization, n = number of mice for each group

We then performed optical mapping on a whole-heart Langendorff preparation. The mapping area was focused on the anterior aspect of the heart ventricle before and after acute isoproterenol (10 nmol/L) perfusion. The concentration of isoproterenol is low to avoid changing APD. Figure 5A shows examples of the optical action potential duration (APD) and intracellular Ca^2+^ transient (CaT) duration (CaTD) maps of WT and CD44^−/−^ mice’s ventricles, at different pacing cycle lengths (PCLs), before and after isoproterenol. The wild-type control and CD44^−/−^ mice ventricles had comparable APD at 80% repolarization (APD_80_) and CaT at 80% repolarization (CaTD_80_) at each PCL. Both the APD_80_ and CaTD_80_ were significantly reduced by isoproterenol at PCL of 80, 100 and 140 ms in the wild-type control and CD44^−/−^ mice ventricles. (Fig. 5A) The coefficient of variation (COV) for either APD_80_ or CaTD_80_ was similar between the wild-type control and CD44^−/−^ mice, suggesting a similar degree of heterogeneity. Figure 5B showed that in the whole-heart preparation, ventricular tachyarrhythmia cannot be induced in any wild-type controls or CD44^−/−^ mice at baseline by regular pacing at constant PCL (S1). After isoproterenol infusion, ventricular tachyarrhythmia was induced in 11 of 14 WT controls and 3 of 13 CD44^−/−^ mice by rapid pacing (*P* < 0.05), respectively. We also assessed the incidence of delayed afterdepolarization (DAD) during or after a long pacing train. Neither WT nor CD44^−/−^ mice exhibited any DAD at baseline. In contrast, 7 of 14 WT mice and 1 of 13 CD44^−/−^ mice developed DAD during or after a long pacing train after isoproterenol infusion (*P* < 0.05). The results suggested CD44 may contribute to ventricular arrhythmias through β-AR although it may not be indispensable for β-AR-related arrhythmogenesis.

### Inducibility of ventricular arrhythmias in heart failure

To evaluate the in vivo contribution of CD44 to ventricular arrhythmias (VAs) in heart failure (HF) associated with β-AR activation, WT and CD44^−/−^ mice were injected with isoproterenol (30 mg/kg per day subcutaneously for 15 days), shown previously to induce HF [20]. VA inducibility was evaluated by intracardiac burst pacing [21]. The electrocardiogram showed the QRS width and QT interval were similar between WT and CD44^−/−^ mice at baseline and with HF. (Additional file 10: Table S1) Additional file 8: Figure S7 shows that 15-d administration of isoproterenol caused a significant increase in left ventricular (LV) diameter and a decrease in LV ejection fraction (EF) in both WT and CD44^−/−^ mice.

**Fig. 6:**
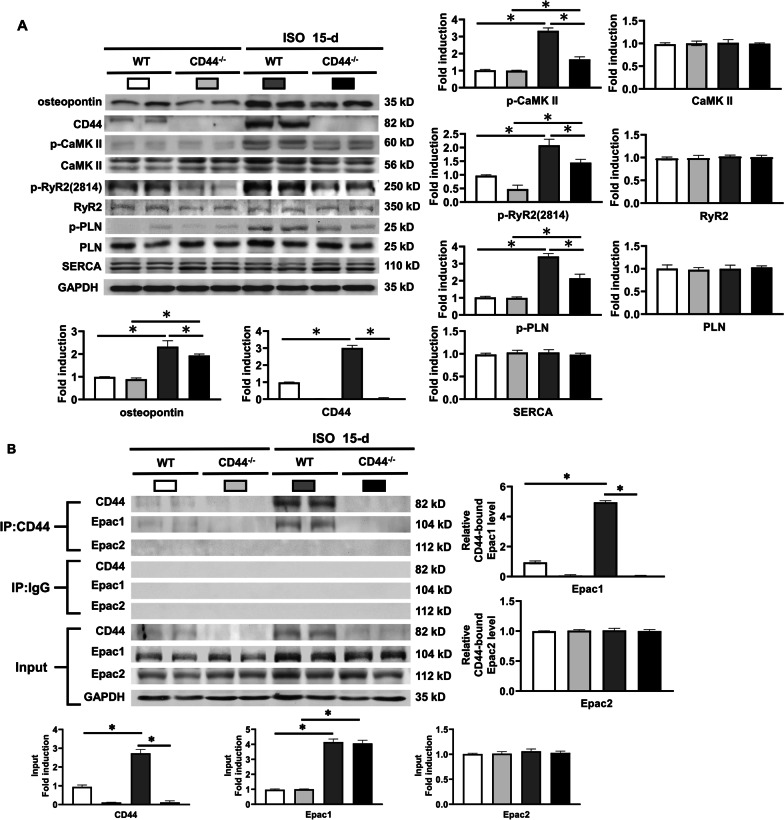

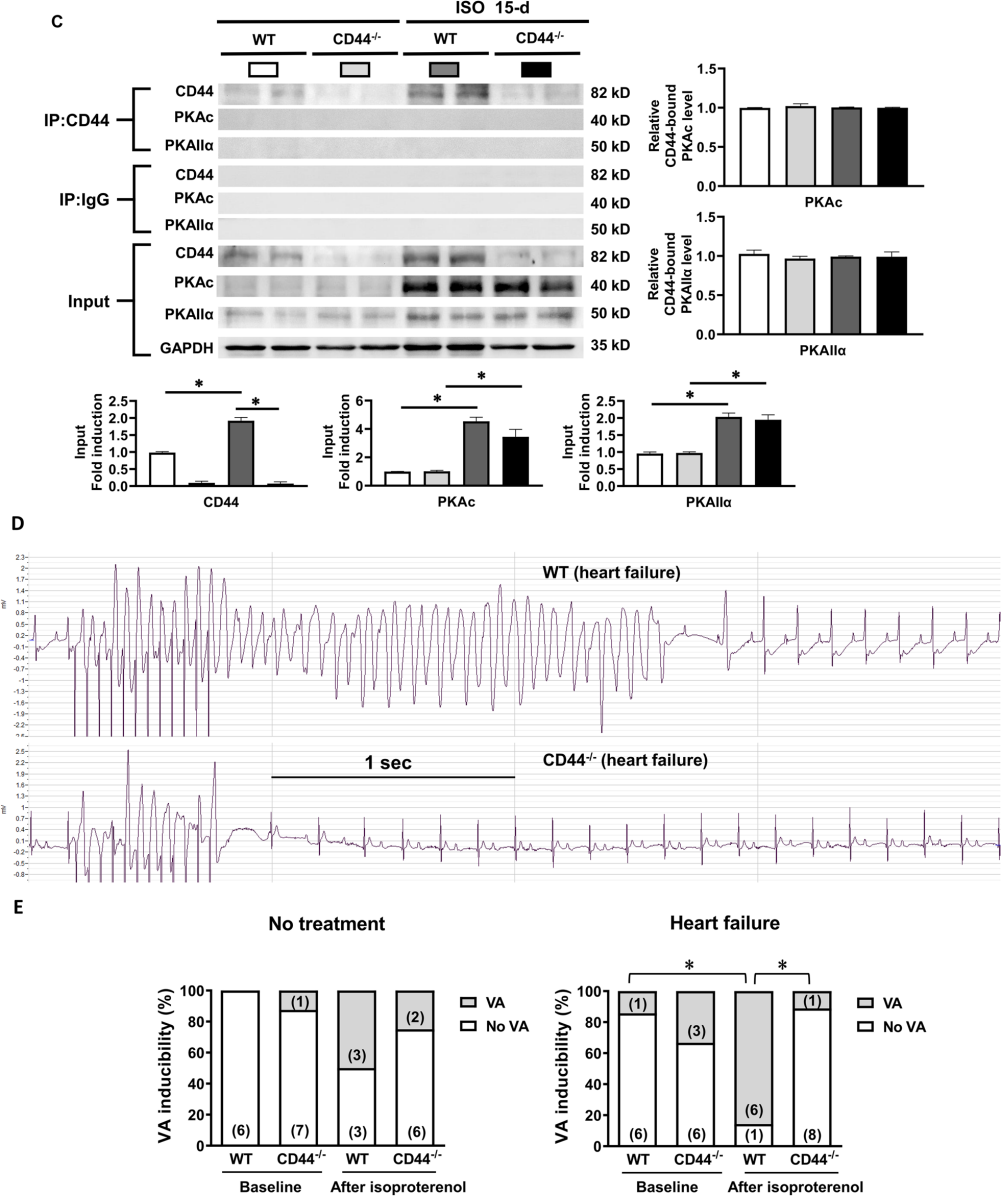
Increased expression of osteopontin, CD44 and Epac and inducibility of ventricular arrhythmias in heart failure mice. **A**–**C** ISO (30 mg/kg per day subcutaneously for 15 days) led to cardiac contractile dysfunction in mice. **A** Representative examples and mean ± SE analysis western blot for osteopontin, CD44, Epac1 and Epac2 in WT and CD44^−/−^ mice treated with ISO. *N* = 4 for each group. *P < 0.05 versus control (WT without ISO) by one-way ANOVA with Bonferroni’s post hoc test. **B** Representative examples and mean ± SE analysis for co-immunoprecipitation of CD44 and control IgG with Epac1 and Epac2. *N* = 3 for each group. **C** Representative examples and mean ± SE analysis for co-immunoprecipitation of CD44 and control IgG with PKAc and PKAIIα. *N* = 3 for each group. In **B** and **C**, the pictures are representation of blots from 3 independent experiments for each. The mean ± SE analysis was for precipitated protein-bound CD44 and Epac1 to precipitated protein ratio and cell input, both of which were quantified to GADPH and normalized to the control level, which was set at 1.0. *P < 0.05 versus control by one-way ANOVA with Bonferroni’s post hoc test. **D** and **E** Inducibility of ventricular arrhythmias (VAs). Inducibility of VAs was tested by intra-cardiac pacing via an internal jugular vein access. A rhythm with more than 5 consecutive ventricular beats after burst pacing for 5 times was considered to be VAs. **D** Representative examples of VAs in WT and CD44^−/−^ mice with heart failure induced by 15-d ISO. All the examples after on-spot single bolus of isoproterenol. **E** Mean ± SE analysis VA inducibility in WT and CD44^−/−^ mice at baseline and after treatment with ISO, and before and after on-spot single bolus of isoproterenol (0.5 mg/kg, i.p.). N = 6–9 mice for each group. *P < 0.05 between groups by Fisher’s exact test. VAs = ventricular arrhythmias, WT = wild-type control mice, CD44^−/−^ = CD44 knock-out mice, ISO = isoproterenol at 30 mg/kg per day subcutaneously for 15 days, (number) = number of the mice

Figure 6 shows that the activation of Ca^2+^-handling proteins and the association between CD44 and Epac1 induced by β-AR activation were comparable between mice treated with 48-h and 15-d isoproterenol. In Fig. 6A, it was shown that the expression of osteopontin was significantly increased in WT and CD44^−/−^ HF mice induced by 15-d isoproterenol. The expression of p-CaMKII, p-RyR2 (Ser-2814) and p-PLN were significantly increased in HF WT mice, which was not noted in CD44^−/−^ mice. Figure 6B shows the association between CD44 and Epac1 was significantly increased in WT HF mice.

Confocal immunofluorescence confirmed the co-localization between CD44 and Epac1 in WT HF mice (Additional file 9: Figure S8). Similar to WT mice treated with 48-d isoproterenol, long-term (15-d) β-AR activation did not cause the association between CD44 and PKAc and PKAIIα in WT HF mice (Fig. 6C).

The VA inducibility was evaluated by intracardiac burst ventricular pacing at baseline and after isoproterenol (0.5 mg/kg, i.p.) injection. Figure 6E shows that at baseline, the VA inducibility was comparably low between WT and CD44−/− mice with or without HF. After a single bolus of isoproterenol during the study, VAs were more easily induced in WT HF mice, and CD44^−/−^ HF mice showed a significantly reduced VA inducibility compared with WT HF mice (12.5% vs. 85.7%, P < 0.05). Taken together, the results suggested that elimination of CD44 is effective for reducing VA inducibility, which may present as a therapeutic target in preventing VAs in HF.

### CD44 is upregulated in human heart failure

**Fig. 7:**
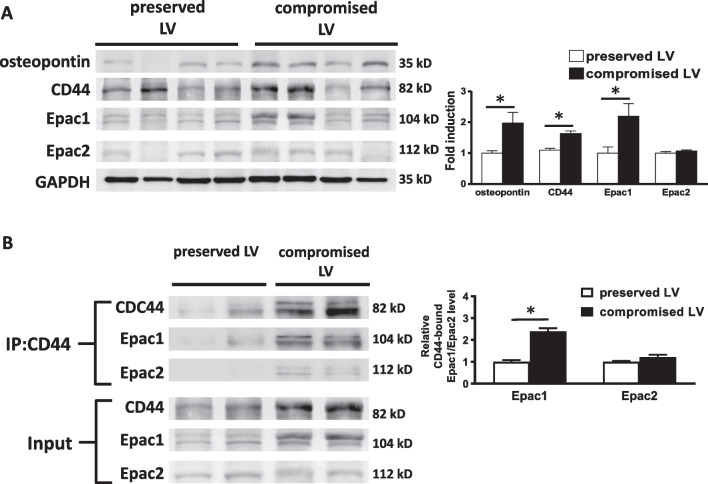
Increased association between CD44 and Epac1 in compromised human heart.1. **A** Representative examples and mean ± SE analysis western blot for osteopontin, CD44, Epac1 and Epac2 in the left ventricle (LV) from patients with preserved and compromised LV contractility. The relative expression of each protein was quantified by densitometry and normalized to GAPDH. The relative expression of each protein was quantified to GAPDH by densimetry and normalized to preserved LV contractility. **B** Representative examples and mean ± SE analysis for co-immunoprecipitation of CD44 and Epac1/Epac2 in human heart. The input reported in **B** is a re-presentation of the 4 middle lanes of the western blot reported in **A**. The mean ± SE analysis was for precipitated protein-bound Epac1 and Epac2 to precipitated protein (CD44) ratio and cell input, both of which were quantified to GADPH and normalized to the group of preserved LV. In **A** and **B**, *N* = 4 for each group. *P < 0.05 between preserved LV and compromised LV by unpaired Student’s t-test. *LV* left ventricle

The expression of CD44 and its interaction with Epac in left ventricle (LV) samples from patients were also evaluated. The patient characteristics are shown in Additional file 11: Table S2. The LV tissue was obtained from patients with severe chronic mitral regurgitation who received mitral valve surgery. The patients were divided to two groups: patients with preserved LV contractility with LV ejection fraction (LVEF) > 60% and patients with compromised LV contractility with LVEF < 50%. As shown in Fig. 7A, compromised LV contractility was associated with increased expression levels of osteopontin, CD44, and Epac1 in LV tissues. Co-immunoprecipitation was performed to evaluate the binding between CD44 and Epacs. The lysates of ventricle tissue were immunoprecipitated using anti-CD44 antibodies. The immunocomplexes were resolved and reblotted with anti-Epac 1/2 or anti-CD44 antibodies. Figure 7B showed that compromised LV was associated with increased expression levels of CD44 and Epac1 and an increased association between these two elements compared with preserved LV. The analysis showed the ratio of CD44-associated Epac1 to CD44 was increased in compromised LV. The results suggested that CD44 may contribute to ventricular arrhythmias in patients with diseased heart. Overall, our findings suggest that CD44 may present a novel therapeutic target in preventing ventricular arrhythmias.

## Discussion

**Fig. 8:**
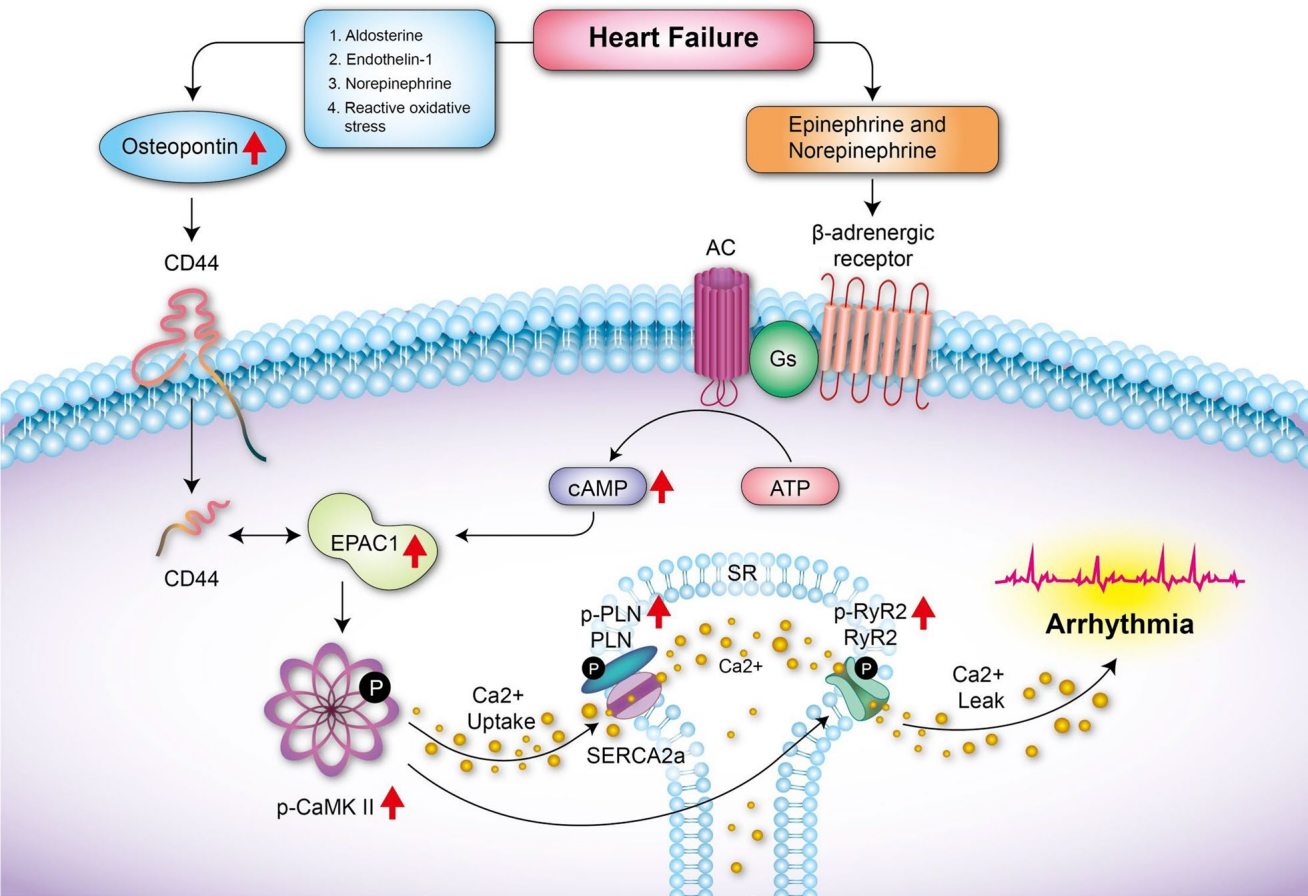
Schema of mechanism for CD44 regulating cardiac β1-adrenergic Epac1-dependent abnormal Ca^2+^ release from SR

In this study, we first demonstrated that CD44 signaling contributes to β-AR-induced activation of RyR2 and PLN through the interaction with Epac1 and activation of CaMKII. Inhibition of CD44 is effective in preventing β-AR-induced Epac1-dependent activation of RyR2 and PLN, which leads to Ca^2+^ leakage from SR and the subsequent VAs. CD44 is upregulated in HF. Eliminating CD44 is effective in reducing VA inducibility in HF mice. CD44 may therefore present a novel therapeutic target for preventing VAs in HF patients. Figure 8 shows the illustrated pathway.

Both Epac1 and Epac2 play physiological and pathological roles in the heart. [4] Both Epac1 and Epac2 have been reported to mediate β-adrenergic receptor-dependent arrhythmias by activating CaMKII. In the heart, the expression level of Epac1 is higher than that of Epac2. Epac1 knockout mice display improved cardiac function during HF induced by the continuous stimulation of β-AR or other forms of stress such as pressure overload [5, 22]. We observed that Epac1, rather than Epac2, was more upregulated in compromised LV tissue than in LV tissue with preserved EF. Upregulation of Epac1, likely a compensation to compromised LV contractility, may contribute to SR Ca^2+^ leakage and the subsequent triggering of arrhythmic activities. Targeting CD44/Epac1 signaling may therefore present an effective strategy in preventing ventricular arrhythmia in HF patients.

CD44 is a transmembrane receptor, with hyaluronan and osteopontin as its ligands. It mediates widespread cellular inflammatory, differentiative, and fibrotic responses in different cell types, including cancer cells, fibroblasts, and myocytes. [23] CD44 is a potential target for preventing fibrosis [24]. The deletion of CD44 protects the heart against angiotensin II-induced cardiac fibrosis in mice; [25] angiontensin II increases TNF-α levels and causes an imbalance between NF-κB and IκB. Thereafter, CD44 in vascular endothelium and inflammatory cells is upregulated and causes aggregation of inflammatory cells, TGF-β1 upregulation, myofibroblast proliferation, and collagen deposition. Recently, we showed that atrial fibrillation (AF) is associated with CD44 upregulation in the atria of patients [13]. We also showed that tachypacing promotes an interaction between CD44 and NOX in myocytes, contributing to atrial arrhythmogenesis, which can be attributed to increased levels of oxidative stress. Tachypacing causes binding between CREB and CD44 in myocytes, which prevents the binding of p-CREB to the promoter of Cav1.2, resulting in reduced inward L-type calcium currents and APD shortening in AF [14]. We also showed that CD44 is involved in atrial fibrosis [26]. CD44 mediates TGF-β1-induced STAT3-dependent upregulation of collagen in atrial fibroblasts. In cardiac-specific TGF-β1-transgenic mice, the administration of neutralizing anti-CD44 antibodies effectively reduces atrial fibrosis and AF inducibility.

Few studies have evaluated the interaction between CD44 and Epac. In mouse articular chondrocytes, hyaluronan fragments activate CD44 crosstalk with cAMP-activated Epac and PKA to exert effects on the NF-κB complex and signaling proteins [27]. In addition, Epac has been shown to be involved in CD44-mediated signaling. In endothelial progenitor cells, CD44 and β2-integrin are homogeneously distributed on the surface. Activation of Epac by 8-CPT results in opposite polarization of the β2-integrin and CD44 on the cell surface, endowing the cells with migratory ability [28]. Furthermore, calcitonin binding to a G protein-coupled receptor activates CD44 transcription, through PKA/extracellular regulated kinase (ERK) and Epac/p38 signaling, in prostate cancer cells [29]. Using co-immunoprecipitation, we provided evidence that CD44 interacts with Epac1 in the heart. Our findings are consistent with the previous studies showing synergic effects between CD44 and Epac and further reveal how CD44 interacts with Epac1 in mediating downstream signaling in myocytes.

Osteopontin is a glycoprotein that is expressed in various cell types, including myocytes and fibroblasts. It has been shown that osteopontin was upregulated by isoproterenol in bone [30]. Osteopontin is increased in HF, according to prior research. Osteopontin contributes to adverse remodelings such as fibrosis in animal models with HF [31]. It has been demonstrated that a number of substances, including aldosterone, endothelin-1, norepinephrine, and reactive oxidative stress, cause osteopontin production in cardiomyocytes [32]. Osteopontin stimulates apoptosis in adult cardiac myocytes via the involvement of CD44. We showed that osteopontin/CD44 signaling likely contributes to cardiac arrhythmias through interaction with β-AR/Epac signaling.

In diseased heart, increased oxidative stress may play an important role in mediating hypertrophy, fibrosis, worsening LV function, and arrhythmia. NOX4 is a major NADPH oxidase isoform expressed in the heart [33]. In one of our previous studies that investigated atrial myocytes, we found that CD44 may also contribute to Ca^2+^-handling protein remodeling in atria as a result of an interaction with NOX4 and increased oxidative stress.

We showed that the expression of CD44 and Epac1 as well as the association between the two proteins were increased by β-AR activation. Increased expression of the two proteins may contribute to the increased association between them. Further analysis showed the ratio of CD44-associated Epac1 to CD44 was also increased by β-AR activation, suggesting the intensity of interaction between CD44 and Epac1 was also likely increased.

As seen in Additional file 8: Figure S7A and B, both WT and CD44^−/−^ mice had significantly lower LVEFs 15 days after receiving isoproterenol, however CD44^−/−^ mice had a tendency to have lower reductions (48.6% vs. 43%, P = n.s.). Although it has been determined that treating RyR calcium leaks is an effective treatment for cardiac contractile dysfunction, [34] other disease processes and signaling cascades, such as the loss of myofibrils and myocytes, cardiac fibrosis, activation of the renin-angiotensin system, and the release of cytokines, in addition to RyR maladaptations and calcium leak, play a role in heart failure and contractile dysfunction [35]. Although the removal of CD44 stops chronic β-AR activation-induced RyR leak, it is yet unknown if doing so will significantly reverse cardiac contractility, which would require further investigation.

Even though we have shown that a single bolus of high-dose isoproterenol causes a brief but non-sustained upregulation in p-RyR2, it remains unclear about the signaling pathways leading to sustained upregulation of p-RyR2 and p-PLN after repeated and prolonged (> 48 h) β-AR stimulation. Our study suggests that upregulated CD44 may contribute to the maladaptation of p-RyR2 and p-PLN. Further investigation will be required to clarify the unknown signaling cascades.

Unexpectedly, Fig. 6D and Additional file 10: Table S1 showed the PR intervals were noticeably longer in CD44^−/−^ HF mice, indicating CD44 may play a role in atrioventricular node function. The underlying mechanism would also need to be clarified through additional research.

In Fig. 7, it was shown that in patients, the interaction between CD44 and Epac2 seemed to be a little increased in compromised LV (P = 0.15). The sample size is too small to draw a conclusion. We speculated that compromised LV in humans presents a more complex condition than excessive activation of adrenergic system, contributing to the interaction between Epac2 and CD44. Thereby, we would like to carefully conclude that β-adrenergic stimulation and osteopontin increase the interaction between Epac1 and CD44, contributing to cardiac arrhythmia. It remains unclear through which mechanism CD44 would interact with Epac2. Meanwhile, its significance may require further investigation.

We showed CD44 mediates β-AR-induced hyperphosphorylation of RyR and PLN, which supposedly results in Ca^2+^ leak from sarcoplasmic reticulum through hyperphosphorylated RyR. Although we have shown that upregulated CD44 decreased L-type calcium currents followed by shortening of APD in rapidly activated atrial myocytes [14], we did not evaluate if inhibiting CD44 would increase L-type calcium currents, which may subsequently contribute to prolonged APD and increased intracellular Ca^2+^ transient. Since β-AR activation by isoproterenol has been known to increase L-type calcium channels and currents independent of CD44 [36], the net effect of isoproterenol and the role of CD44 during β-AR activation on them is likely complex and beyond the scope of the study.

### Limitation

Since the studies were conducted using cell lines and mice administered with high-dose isoproterenol to activate of β-AR, rather than in an animal model of diseased or failing heart, we only demonstrated the molecular mechanism by which CD44 participates in β-AR- and Epac-1-induced Ca^2+^-handling abnormalities and triggered arrhythmias. It would need further experimental studies to verify if blocking CD44 is an effective strategy for treating or preventing cardiac arrhythmias in animal model of heart disease.

## Conclusions

This study showed that osteopontin/CD44 signaling is likely arrhythmogenic through direct interaction between CD44 and Epac1 to regulate β-adrenergic Epac1-dependent hyperphosphorylation of RyR2 and PLN. CD44 is also upregulated in the ventricles of patients with compromised hearts. Our findings may provide new therapeutic insights to prevent cardiac arrhythmias and sudden death in patients with diseased hearts.

## Supplementary Information

**Additional file 1.** Methods.

**Additional file 2: Figure S1.** Representative examples and mean ± SE analysis western blot for (p-)RyR and (p)-PLN within 12 h after single bolus of ISO in WT and CD^−/−^ mice. The relative expression of each protein was quantified to GAPDH by densitometry and normalized to the control. *N* = 4 for each group. *P < 0.05 versus control (WT and CD44^−/−^ mice without ISO) by one-way ANOVA with Bonferroni’s post hoc test. WT = wild-type control mice, CD44^−/−^ = CD44 knock-out mice, ISO = isoproterenol at 30 mg/kg

**Additional file 3: Figure S2.** Reverse co-immunoprecipitation for CD44 and Epac1. Representative examples and mean ± SE analysis for co-immunoprecipitation of CD44 with Epac1 and reverse co-immunoprecipitation of Epac1 with CD44 in WT and CD44^−/−^ mice treated with 48-h ISO. The pictures are representation of blots from 3 independent experiments for each. the mean ± SE analysis was for precipitated protein-bound CD44 and Epac1 to precipitated protein ratio and cell input, both of which were quantified to GADPH and normalized to the control (WT without ISO) level, which was set at 1.0. *N* = 3 for each group. *P < 0.05 versus control (WT and CD44^−/−^ no ISO) by one-way ANOVA with Bonferroni’s post hoc test. WT = wild-type control mice, CD44^−/−^ = CD44 knock-out mice, ISO = isoproterenol at 30 mg/kg per day subcutaneously.

**Additional file 4: Figure S3.** Efficacy of siRNA knockdown and plasmid transfection. Representative examples (Upper) and mean ± SE analysis (Lower) western blot for (A) CD44, (B) Epac1 and (C) Epac2 in HL-1 myocytes transfected with CD44 siRNA, Epac1 siRNA or Epac2 siRNA respectively and (D) CD44 in HL-1 myocytes transfected with wild-type CD44 cDNA-containing plasmids. The relative expression of each protein was quantified to GAPDH by densitometry and normalized to GAPDH. n = 3 for each group. *p < 0.05 versus control by one-way ANOVA with Bonferroni’s post hoc test.

**Additional file 5: Figure S4.** Effects of osteopontin, β-AR and Epac activation on Epac. Representative examples and mean ± SE analysis western blot for (A) Epac1 and (B) Epac2 in HL-1 myocytes treated with osteopontin, isoproterenol, 8-CPT-cAMP or Epac siRNAs. The relative expression of each protein was quantified to GAPDH by densitometry and normalized to GAPDH. *n* = 4 for each group. *P < 0.05 versus control by one-way ANOVA with Bonferroni’s post hoc test. ISO = isoproterenol

**Additional file 6: Figure S5.** Proximity ligation assay and co-localization of CD44 and Epac1 in HL-1 myocytes. **A.** Representative con-focal images of proximity ligation assay with WGA (green color), which was used to mark cell membrane in HL-1 myocytes. The red color (arrows) indicates the association between CD44 and Epac1. Representative examples of z-stacking mode in HL-1 myocytes with (c, d) and without (a, b) treatment with isoproterenol. **B.** Representative con-focal images of CD44 (upper, green color), Epac1 (middle, red color) and co-localization of both (lower, yellow color) and mean ± SE analysis of co-localization in HL-1 myocytes treated with isoproterenol. The relative fluorescence of co-localization was normalized to control as 1.0. *n* = 4 images per group. *P < 0.05 versus control by unpaired Student t-test. Bar = 10 μm. ISO = isoproterenol, WGA = wheat germ agglutinin.

**Additional file 7: Figure S6.** Representative examples of Ca^2+^ waves in ventricular myocytes from WT mice pretreated with flecanide (10 μM) for 20 min, tocanide (50 μM) or tetracine (50 μM) for 10 min and after isoproterenol and 3-Hz electrical stimulation. The results were confirmed from 9—18 cells for each group. WT = wild-type mice, ISO = isoproterenol.

**Additional file 8: Figure S7.** Examples of M-mode echocardiographic images (A) and mean ± SE analysis for LVEF (B) of the heart from WT and CD44^−/−^ mice at baseline and after treatment with ISO. *N* = 5 for each group, *p < 0.05 versus control (WT without ISO) by one-way ANOVA with Bonferroni’s post hoc test. WT = wild-type control mice. CD44^−/−^ = CD44 knock-out mice, WT = wild-type control mice, ISO = Isoproterenol at 30 mg/kg/day subcutaneously for 15 days. LVEF = left ventricular ejection fraction.

**Additional file 9: Figure S8.** Co-localization of CD44 and Epac1 in heart failure mice. Representative confocal images of CD44 (upper, green color), Epac1 (middle, red color) and co-localization of both (lower, yellow color) and mean ± SE analysis of co-localization in WT and CD44^−/−^ mice heart at baseline and after treatment with ISO. The relative fluorescence of co-localization was normalized to WT as 1.0. *N* = 4 per group. *P < 0.05 versus control by one-way ANOVA with Bonferroni’s post hoc test. WT = wild-type control mice, CD44^−/−^ = CD44 knock-out mice, ISO = isoproterenol at 30 mg/kg per day subcutaneously for 15 days, HF = heart failure.

**Additional file 10: Table S1.** The electrocardiography parameters in wild-type and CD44^−/−^ mice at baseline and with heart failure

**Additional file 11: Table S2.** Characteristics of the patients

## Data Availability

Not applicable.

## Abbreviation

APD: Action potential duration
AF: Atrial fibrillation
β-AR: β-Adrenergic receptor
CaMKII: Ca^2+^/calmodulin-dependent protein kinase II
CaSpF: Ca^2+^ spark frequency
CaT: Ca^2+^ transient
CaTD: Ca^2+^ transient duration
CD44^−^^/^^−^: CD44 knockout
COV: Coefficient of variation
8-CPT-cAMP: 8-(4-Chlorophenylthio) adenosine-3’,5’-cyclic monophosphate
cAMP: Cyclic adenosine monophosphate
DAD: Delayed afterdepolarization
ERK: Extracellular regulated kinase
Epac: Exchange proteins directly activated by cAMP
HF: Heart failure
LVEF: Left ventricular ejection fraction
NOX: NADPH oxidase
PCL: Pacing cycle length
PLN: Phospholamban
PKA: Protein kinase A
RyR: Ryanodine receptor
SERCA: Sarcoendoplasmic reticulum calcium ATPase
siRNA: Small interfering RNA
NCX: Sodium-calcium exchanger
VA: Ventricular arrhythmia
