# Supplementary material for: CD44 regulates Epac1-mediated β-adrenergic-receptor-induced Ca2+-handling abnormalities: implication in cardiac arrhythmias
Source: J Biomed Sci. 2023 Jul 14;30:55. doi: 10.1186/s12929-023-00944-0 (PMC10347873; doi:10.1186/s12929-023-00944-0)
Supplement: Supplementary file 1 — Additional file 1. Methods. [file 12929_2023_944_MOESM1_ESM.doc]

**Supplementary Methods**

**Animals**

**CD44 ^-/-^ mice**

Wild-type C57BL/6 and CD44 knockout (CD44^-/-^) mice (Jax#005085, B6.129(Cg)-Cd44^tm1Hbg^/J) were purchased from the Jackson Laboratory. The genotypes of the mice were confirmed by diagnostic polymerase chain reaction (PCR). The CD44^-/-^ mice present low constitutive level of CD44 by western blotting, which show no response to CD44-activating agents or stress as described previously [1, 2].

To study β-AR-induced arrhythmias, male mice (8-12 weeks old) were administrated isoproterenol daily bolus at a concentration of 30 mg/kg subcutaneously. Thereafter, mice were sacrificed 12 h, 24 h, 48 h and 15 d after the first bolus of isoproterenol. The mice were anesthetized with Zoletil® (50 mg/kg) and xylazine (10 mg/kg), intraperitoneally. The mice 15 d after isoproterenol were subject to echocardiography and electrocardiogram. After confirming a fully anaesthetized state (e. g. no response to toe pinching), the heart was isolated. The animal study was approved by the Institutional Animal Care and Use Committee of Chang-Gung Memorial Hospital.

**Myocyte Isolation**

After the mice were anesthetized as described in the “**CD44-/- mice**” section, the excised hearts were cannulated and retrogradely perfused with nominally Ca^2+^ free HEPES-buffered Tyrode’s solution. The perfusate was oxygenated and kept at 37 ± 0.2 °C for 5 min. The perfusate was subsequently transferred into the same solution containing 0.5 mg/mL collagenase (type II, Worthington Biochemical, Lakewood, NJ) and 0.05 mg/mL protease (type XIV, Sigma-Aldrich, St. Louis, MO). After 30 min, the residual enzymatic solution was removed by perfusion with Ca^2+^ free HEPES-buffered solution supplemented with bovine serum albumin (0.5 mg/mL) and 0.05 mmol/L CaCl_2_. Ventricular myocytes were isolated from digested ventricles by gentle mechanical dissociation and the myocytes were then stored in Tyrode solution containing 0.2 mmol/L Ca^2+^.

**HL-1 myocytes**

HL-1 myocytes were maintained in Claycomb medium as described previously.[2] In all Western blot and co-immunoprecipitation cell experiments, HL-1 myocytes were treated with chemicals, peptide or plasmids as indicated for 24 h.

**Expression vectors, small interfering RNA (siRNA), and transfection**

Wild-type CD44 cDNA-containing plasmids were bought from Genediscovery Biotechnology (Taiwan) and subcloned into a pcDNA3.1(−)/Myc-His vector. His-tag CD44 cDNA-containing plasmids were kindly provided from Professor Jia-Lin Lee, National Tsing-Hua University, Taiwan. The vectors were transfected into HL-1 myocytes using Lipofectamine 2000 (Thermo Fisher Scientific Inc, Waltham, MA, USA) and used for experiments 24 h after transfection. Chemically synthesized siRNAs for CD44, Epac1 and Epac2 as well as their control siRNAs were purchased from Dharmacon (Lafayette, CO) and transfected into HL-1 myocytes using DharmaFECT 1 (Dharmacon) according to the manufacturer's instructions. They included on target siRNA-smart pools with four sequences. A concentration of 200 nmol per well was added in a 4-well dish as previously described [2].

**Western blotting**

Western blot was performed as previously described [2]. Briefly, an equal amount of protein in sodium dodecyl sulfate-polyacrylamide gel electrophoresis (SDS-PAGE) sample buffer was sonicated and subjected to electrophoresis on 8% SDS polyacrylamide gels. After being transferred to PVDF membranes (Stratagene, Netherlands), proteins were incubated with primary antibodies. Signals were detected by UVP (Analytik-Jena, Jena, Thuringia) and quantified using the software Image Gauge (Fuji film, Tokyo, Japan). Signal bands were analyzed with GAPDH as a reference.

**Co-immunoprecipitation**

The lysates from HL-1 myocytes or heart tissue were harvested with lysis buffer [25 mmol/L Tris-HCl (pH 7.6), 0.3 mmol/L NaCl, 1.5 mmol/L MgCl_2_, 0.2 mmol/L EDTA, 0.5% Nonidet P-40, and 0.5 mmol/L dithiothreitol] and immunoprecipitated with anti-CD44 antibodies for 2 h at 4 °C. Following incubation with 50 µL Protein A-Sepharose for 1 h at 4 °C, the beads were collected, washed three times, and resuspended in SDS sample buffer. The immunocomplexes were resolved by SDS-PAGE and processed by western blotting. Reverse co-immunoprecipitation was performed using anti-Epac1, anti-Epac2, anti-PAKIIα and anti-PKAc to precipitate the lysates.

**Proximity ligation assay**

Red Starter Duolink kit (Sigma-Aldrich, Sweden) was selected for this experiment and performed following the manufacturer's instructions. Briefly, HL-1 myocytes were pre-treated with osteopontin (100 ng/mL), isoproterenol (1 µmol/L) or 8-CPT-cAMP (100 µmol/L) for 24 h and then incubated in the blocking buffer (provided with the kit) for 60 min at 37 °C in a humidified chamber. The cells were then incubated with primary antibodies for 1 h at room temperature, washed in Buffer A (supplied with the kit) 3 times for 5 min, and incubated with the PLA probes for 1 h at 37 °C. Ligation reaction was conducted at 37 °C for 30 min. The cells were then incubated with the amplification mix for 100 min at 37 °C. After being washed with Buffer B (supplied with the kit), the cells were mounted using the mounting media provided in the kit. Images were captured with confocal microscope. Red indicated the interaction between CD44 and Epac1, which was quantified as the color-occupied area divided by the nucleic area in each cell. For each analysis, at least five random fields were chosen to observe >10 cells.

**Materials and antibodies**

8-CPT-cAMP, Rp-8-CPT-cAMPS and ICI-118,551 was purchased from Abcam (Cambridge, MA), BIOLOG (Germany) and MedChemExpress (NJ) respectively. Osteopontin was purchased from R&D systems (Minneapolis, MN). Antibodies against osteopontin, CD44, sarcoendoplasmic reticulum calcium ATPase (SERCA), phospholamban (PLN), phospho-PLN(p-PLN) (in Figure 2A-C), sodium-calcium exchanger (NCX), Ca^2+^/calmodulin-dependent protein kinase IIδ (CaMKIIδ), and phospho-CaMKIIδ (p-CaMKIIδ) were purchased from Abcam (Cambridge, MA).

Antibodies against RyR2 were purchased from Proteintech Group Inc. (IL). Antibodies against phospho-RyR2 (p-RyR2, Ser2808 and p-RyR2, Ser2814) and p-PLN (in Figure 1A and Figure 2D and E) were purchased from Badrilla (Leeds, UK). Antibodies against oxidized-CaMKII (ox-CAMKII, Met281/282) were purchased from GeneTex Inc. (Irvine, CA). Antibodies against protein kinase A catalytic subunit (PKAc), protein kinase A regulatory IIα (PKAIIα), and GAPDH were purchased from Santa Cruz (CA). The anti-6-His tag antibody was purchased from Fortis Life Sciences (Waltham, MA)

**Immunocytochemical analysis**

Immunocytochemical analysis of heart tissue and HL-1 cells were performed with primary antibodies against CD44 (Abcam, catalog ab6124) and Epac1 (Abcam, catalog ab109415). At the end of the experiments, tissue and cells were fixed with 4% paraformaldehyde, blocked with 2% BSA in PBST for 30 min, and then incubated with primary antibodies for 1 h. Subsequently, the tissue and cells were incubated with Alexa-488-conjugated (green) (Abcam, catalog ab150113) and Cy3-conjugated (red) (abcam, catalog ab97075) secondary antibodies. Nuclei were visualized by 4,6-diamidino-2-phenylindole (DAPI) staining (Invitrogen, catalog P36935, Waltham, MA, USA). Fluorescence overlapping areas were observed with a confocal microscope (Leica, Confocal TCS SP8XL, Wetzlar and Mannheim, Germany) at the Microscope Core Laboratory of Chang Gung Memorial Hospital.

**Generation of neutralizing anti-CD44 blocking monoclonal antibody**

Rat anti-mouse CD44 blocking antibody recognizing the HA binding domain of CD44 was generated as described elsewhere and used for blocking CD44-related effects[2-6]. This antibody was used for blocking CD44-related effects. For the study of Ca^2+^ spark frequency and Ca^2+^ waves, isolated cardiomyocytes were pretreated with anti-CD44 blocking antibody 2 h before pacing 30 min.

**Line Scan Confocal Microscopy and Confocal Ca^2+^ Imaging**

Ca^2+^ spark measurement was performed as previous described.[2] Briefly, HL-1 myocytes were loaded with 10 μmol/L Fluo-4 AM (Molecular Probes, Eugene, OR, USA) for 30 min. To record Ca^2+^ sparks, a line scan mode was used. After sequential scanning, a two-dimensional image of 512 × 1000 lines was generated and stored for offline analysis. Spontaneous Ca^2+^ sparks were acquired along the longitudinal axis of the myocyte for 10 s after they were treated with isoproterenol at 1 µmol/L for 15 min and with/without CD44 plasmid, CD44 siRNA or CD44 blocking antibody.

The isolated ventricular cardiomyocytes were harvested and stored in oxygenated Tyrode’s solution. For calcium indicator staining, 10 μL of Fluo-4 AM stock solution was added to 1 mL cell suspension (final Fluo-4 AM concentration of 10 μmol/L). The isolated ventricular cardiomyocytes were incubated at room temperature for 20 min to allow for complete de-esterification of AM esters. A Leica SP8 scanning system (Leica Microsystems, Buffalo Grove, IL, USA) equipped with a 40× oil immersion lens (NA = 1.3) was used for confocal imaging of Ca^2+^ fluorescence. Fluo-4 was excited by the 488-nm line of an argon laser, and the emission signals were collected through a 505- to 550-nm bandpass filter. For recording of Ca^2+^ transients/sparks, a line scan mode was used. After sequential scanning, a two-dimensional image of 512 × 1000 lines was generated and stored for offline analysis. Ca^2+^ transients were elicited by field stimulation through a pair of platinum electrodes, with a 2 ms suprathreshold square wave voltage pulse delivered by the electrical stimulator at 3 Hz for 30 s to allow a steady-state condition to be reached. Spontaneous Ca^2+^ sparks were acquired in quiescent cells after steady state stimulation at a rate of 3 Hz for 30 s before and after treatment with isoproterenol at 1 µmol/L for 15 min and with/without CD44 blocking antibody.

**Whole Heart Langendorff Preparation**

The heart was harvested immediately and cannulated through the aorta after the mice were anesthetized. Blood was flushed out by injecting 30 mL cardioplegic solution composed of 134 mmol/L NaCl, 15 mmol/L KCl, 20 mmol/L NaHCO_3_, 0.9 mM NaH_2_PO_4_, 1.8 mmol/L CaCl_2_, 0.5 mmol/L MgSO_4_, and 5.5 mmol/L glucose. Subsequently, the heart was cannulated to a Langendorff apparatus and perfused with oxygenated Tyrode’s solution heated to 38.0 °C. The composition of Tyrode’s solution was 134 mmol/L NaCl, 4.5 mmol/L KCl, 0.5 mmol/L MgCl_2_, 2 mmol/L NaH_2_PO_4_, 23 mmol/L NaHCO_3_, 1.8 mmol/L CaCl_2_, and 5.5 mmol/L glucose, equilibrated with 95% O_2_ and 5% CO_2_ to maintain a pH of 7.4.

**Optical Mapping for Whole-Heart Electrophysiology**

We performed simultaneous optical mapping of the membrane potential (V_m_) and Ca_i_ for the mice ventricles using techniques similar to that reported elsewhere. The perfusate was maintained at 38.0 °C with a flow rate of approximately 2 mL/min to 5 mL/min to keep a constant perfusion pressure of 110 cm H_2_O. After 30 min of stabilization, 3.0 mmol/L calcium-sensitive dye (Rhod-2; Life Technologies, Carlsbad, CA, USA) and 10 mmol/L voltage-sensitive dye (RH237; Life Technologies) were added to the perfusate. The heart was washed for 30 min, followed by the addition of 20 mmol/L excitation–contraction uncoupler (blebbistatin; Tocris Bioscience, Minneapolis, MN, USA). Subsequently, the tissue was excited with a Halogen lamp at a wavelength of 532 ± 45 nm. The fluorescence signal was collected by two fast-speed charge-coupled device cameras (BrainVision, Tokyo, Japan). The voltage fluorescence signal was acquired by one camera through a 710-nm long-pass filter, whereas the calcium fluorescence signal was acquired by another camera through a 585- to 620-nm bandpass filter. Fluorescence images were captured at a frame rate of 2 ms/frame, 100 × 100 pixels and a spatial resolution of 0.35 × 0.35 mm^2^/pixel for 4 s. A pair of bipolar electrodes were placed on the RV apex, and the pacing stimuli were 2 ms pulse widths by 2.5 times the diastolic pacing threshold.

**In Vivo inducibility of ventricular arrhythmias**

The programmed intracardiac stimulation was performed in wild-type control and CD44^-/-^ mice with and without 15-d administration of isoproterenol.[7] Briefly, an 1.1F octapolar catheter (FTS-1113A-0518, Transonic Systems Inc. Ithaca, NY) was inserted into right ventricle via the right internal jugular vein. The mice ventricle was subject to 12 beats of burst pacing with a pacing cycle length of 50 ms for five times to evaluate the inducibility of ventricular arrhyhthmias (VAs). The inducibility of VAs was evaluated at baseline and after isoproterenol (0.5 mg/kg, i.p.) injection when the heart rate reached the highest rate. An episode of more than five consecutive ventricular beats was considered to be VA.

**Patients**

Left ventricle (LV) specimens were obtained from eight patients with primary severe mitral regurgitation during mitral valve surgery. The patients were recruited at Chang-Gung Memorial Hospital in Linkou, Taiwan. The study protocol complied with the ethical guidelines of the 1975 Declaration of Helsinki and was approved by the Chang-Gung Medical Foundation Institutional Review Board.

**Statistical Analysis**

Data are expressed as mean ± standard error (SE). For western blot analysis, unpaired Student’s *t*-test was used for independent samples between two groups. One-way analysis of variance (ANOVA) followed by Bonferroni post hoc tests were used for multiple-group comparisons. For Ca^2+^ spark frequency and Ca^2+^ waves, unpaired Student’s *t*-test was used between two groups. Two-way ANOVA followed by Bonferroni post hoc tests were used for action potential duration and Ca^2+^ transient duration analysis. The inducibility of ventricular arrhythmias and delayed afterdepolarizations were calculated by Fisher’s exact test. For patient characteristics and the results from echocardiography and electrocargram, mean ± SE for continuous variables and percent for categorical variables are presented. Continuous variables were calculated by Student’s *t*-test and gender differences were calculated by Chi-Squared test. *P* < 0.05 was considered significantly different.

1. Hasib A, Hennayake CK, Bracy DP, Bugler-Lamb AR, Lantier L, Khan F, Ashford MLJ, McCrimmon RJ, Wasserman DH, Kang L: **CD44 contributes to hyaluronan-mediated insulin resistance in skeletal muscle of high-fat-fed C57BL/6 mice**. *Am J Physiol Endocrinol Metab* 2019, **317**(6):E973-E983.

2. Chen WJ, Chang SH, Chan YH, Lee JL, Lai YJ, Chang GJ, Tsai FC, Yeh YH: **Tachycardia-induced CD44/NOX4 signaling is involved in the development of atrial remodeling**. *J Mol Cell Cardiol* 2019, **135**:67-78.

3. Li Y, Jiang D, Liang J, Meltzer EB, Gray A, Miura R, Wogensen L, Yamaguchi Y, Noble PW: **Severe lung fibrosis requires an invasive fibroblast phenotype regulated by hyaluronan and CD44**. *J Exp Med* 2011, **208**(7):1459-1471.

4. Zheng Z, Katoh S, He Q, Oritani K, Miyake K, Lesley J, Hyman R, Hamik A, Parkhouse RM, Farr AG *et al*: **Monoclonal antibodies to CD44 and their influence on hyaluronan recognition**. *J Cell Biol* 1995, **130**(2):485-495.

5. Chang SH, Yeh YH, Lee JL, Hsu YJ, Kuo CT, Chen WJ: **Transforming growth factor-beta-mediated CD44/STAT3 signaling contributes to the development of atrial fibrosis and fibrillation**. *Basic Res Cardiol* 2017, **112**(5):58.

6. Chang SH, Chan YH, Chen WJ, Chang GJ, Lee JL, Yeh YH: **Tachypacing-induced CREB/CD44 signaling contributes to the suppression of L-type calcium channel expression and the development of atrial remodeling**. *Heart Rhythm* 2021, **18**(10):1760-1771.

7. Sovari AA, Iravanian S, Dolmatova E, Jiao Z, Liu H, Zandieh S, Kumar V, Wang K, Bernstein KE, Bonini MG *et al*: **Inhibition of c-Src tyrosine kinase prevents angiotensin II-mediated connexin-43 remodeling and sudden cardiac death**. *J Am Coll Cardiol* 2011, **58**(22):2332-2339.
