# Supplementary figures and images for: CD44 regulates Epac1-mediated β-adrenergic-receptor-induced Ca2+-handling abnormalities: implication in cardiac arrhythmias
Source: J Biomed Sci. 2023 Jul 14;30:55. doi: 10.1186/s12929-023-00944-0 (PMC10347873; doi:10.1186/s12929-023-00944-0)

A

WT

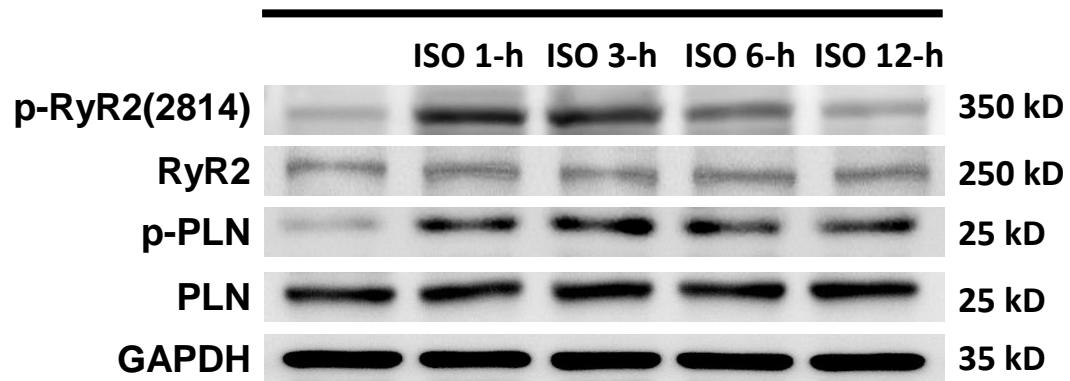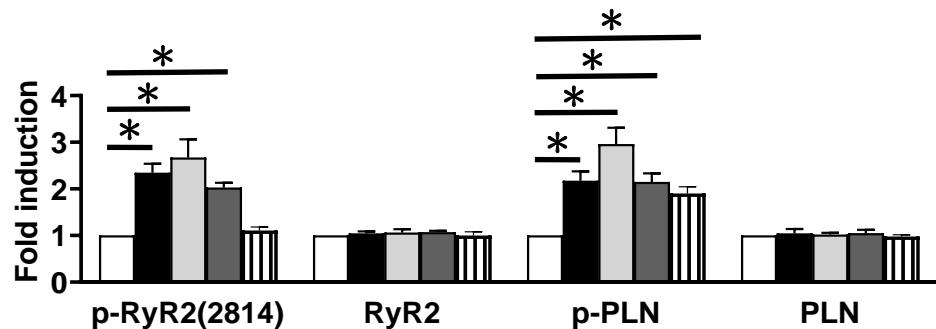

B

CD44<sup>-/-</sup>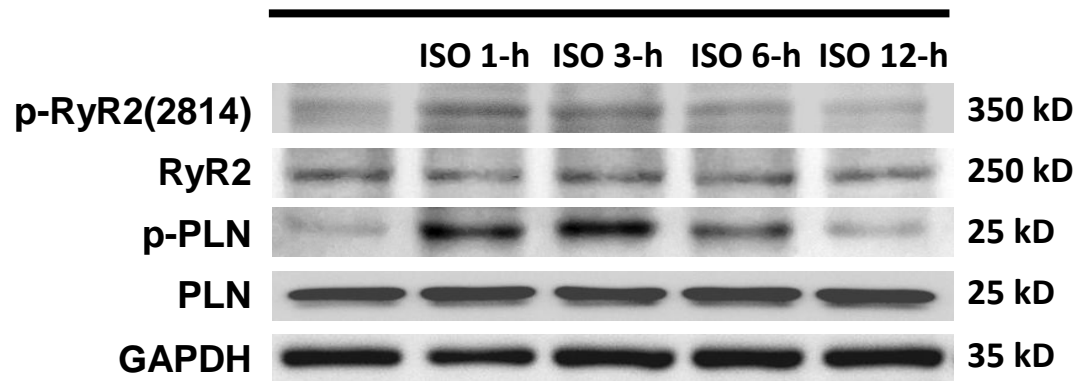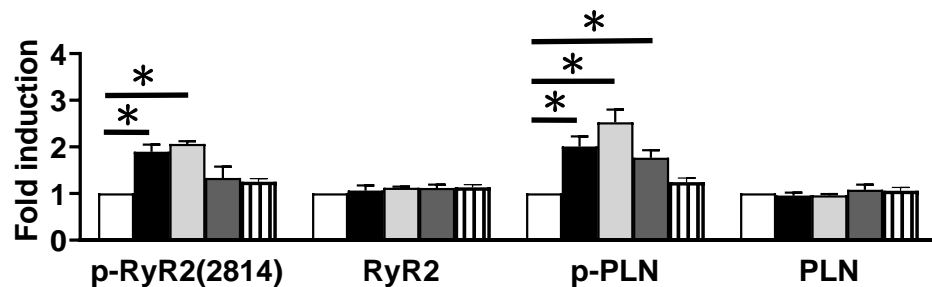

Supplement: Supplementary file 2 — Additional file 2: Figure S1. Representative examples and mean ± SE analysis western blot for (p-)RyR and (p)-PLN within 12 h after single bolus of ISO in WT and CD−/− mice. The relative expression of each protein was quantified to GAPDH by densitometry and normalized to the control. N = 4 for each group. *P < 0.05 versus control (WT and CD44−/− mice without ISO) by one-way ANOVA with Bonferroni’s post hoc test. WT = wild-type control mice, CD44−/− = CD44 knock-out mice, ISO = isoproterenol at 30 mg/kg [file 12929_2023_944_MOESM2_ESM.pdf]

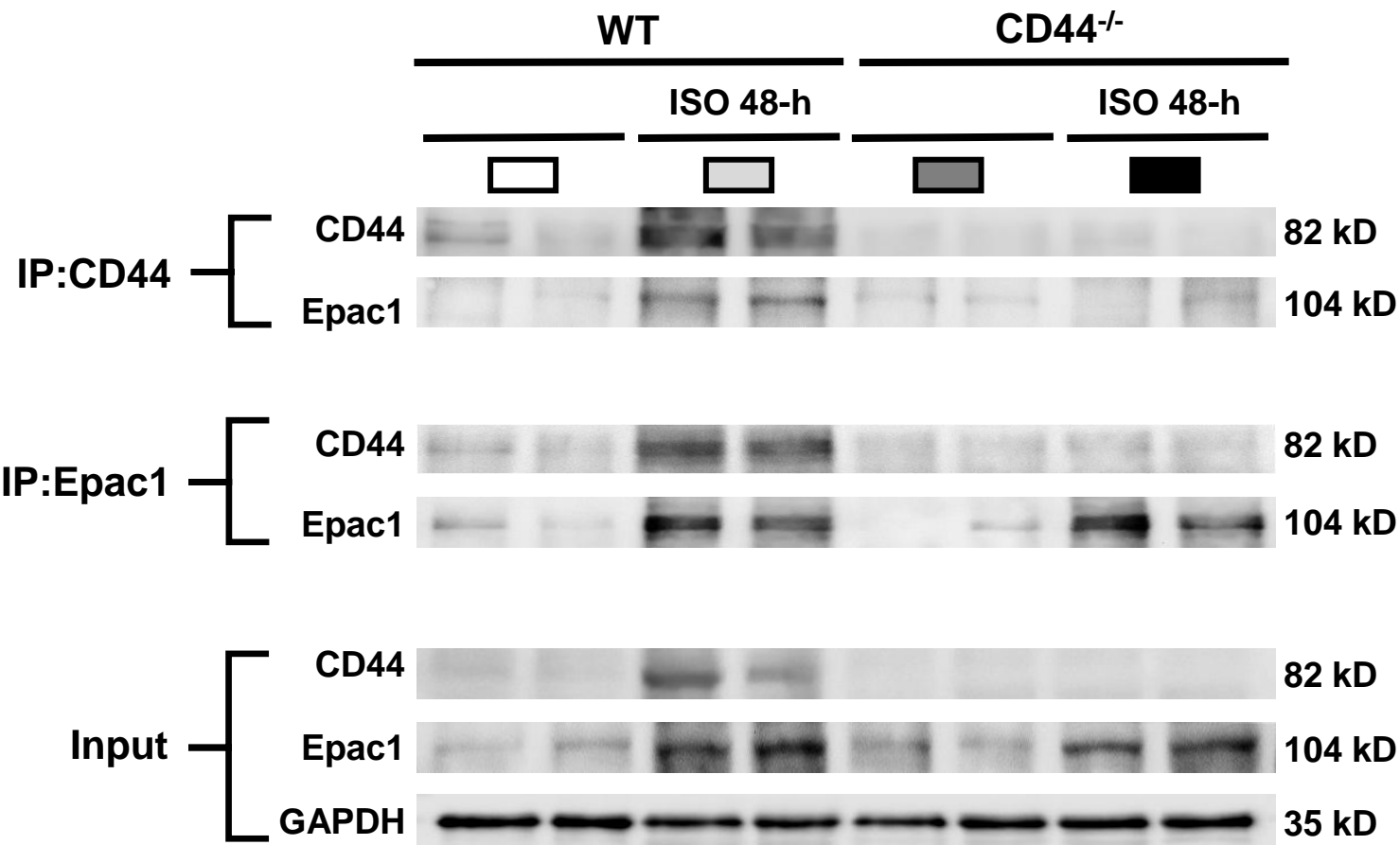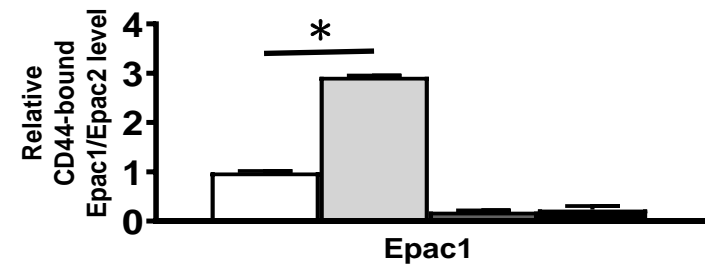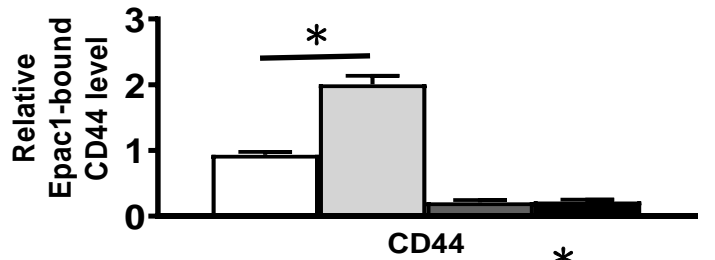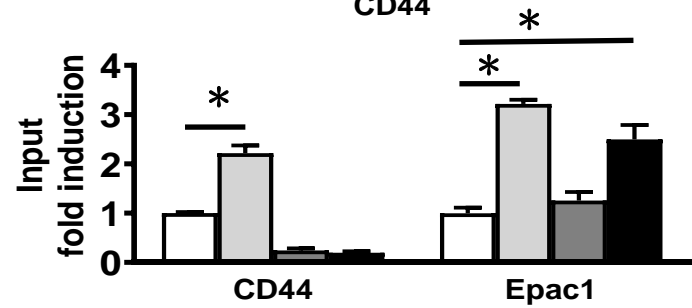

Supplement: Supplementary file 3 — Additional file 3: Figure S2. Reverse co-immunoprecipitation for CD44 and Epac1. Representative examples and mean ± SE analysis for co-immunoprecipitation of CD44 with Epac1 and reverse co-immunoprecipitation of Epac1 with CD44 in WT and CD44−/− mice treated with 48-h ISO. The pictures are representation of blots from 3 independent experiments for each. the mean ± SE analysis was for precipitated protein-bound CD44 and Epac1 to precipitated protein ratio and cell input, both of which were quantified to GADPH and normalized to the control (WT without ISO) level, which was set at 1.0. N = 3 for each group. *P < 0.05 versus control (WT and CD44−/− no ISO) by one-way ANOVA with Bonferroni’s post hoc test. WT = wild-type control mice, CD44−/− = CD44 knock-out mice, ISO = isoproterenol at 30 mg/kg per day subcutaneously. [file 12929_2023_944_MOESM3_ESM.pdf]

**A**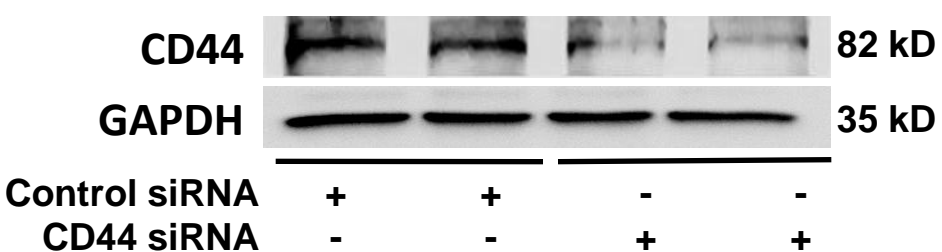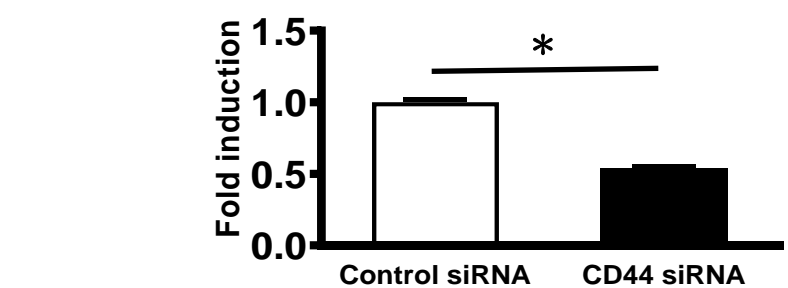**B**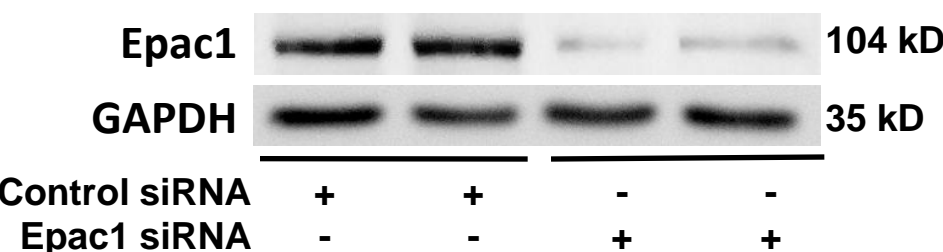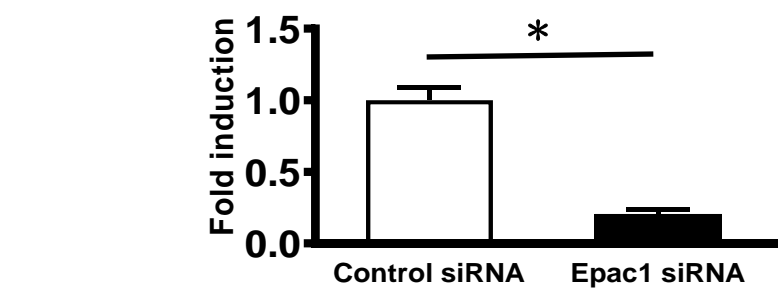**C**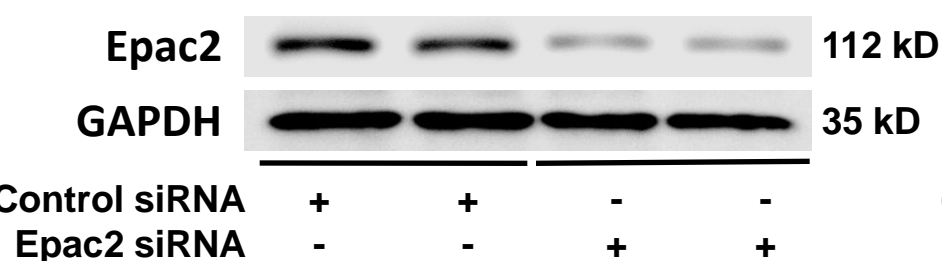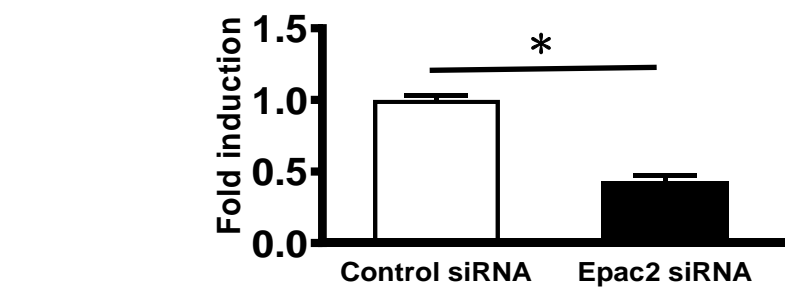**D**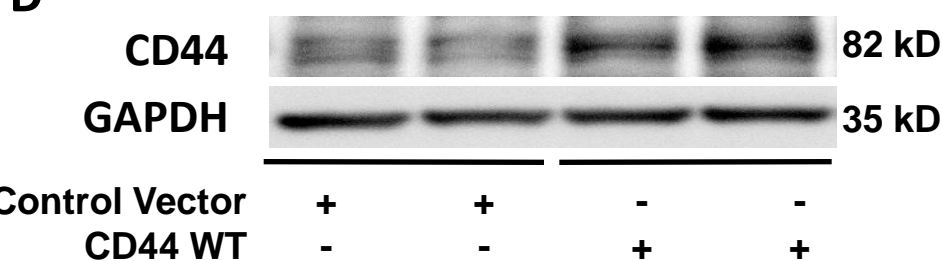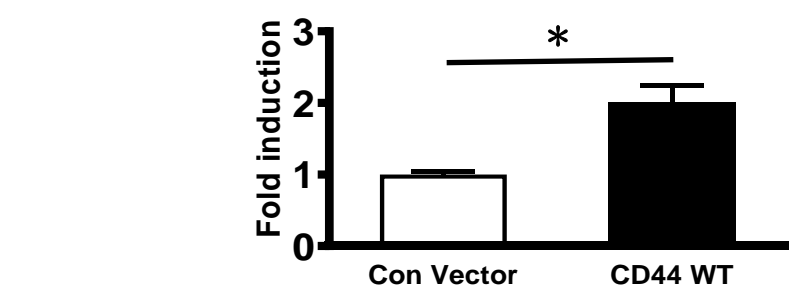

Supplement: Supplementary file 4 — Additional file 4: Figure S3. Efficacy of siRNA knockdown and plasmid transfection. Representative examples (Upper) and mean ± SE analysis (Lower) western blot for (A) CD44, (B) Epac1 and (C) Epac2 in HL-1 myocytes transfected with CD44 siRNA, Epac1 siRNA or Epac2 siRNA respectively and (D) CD44 in HL-1 myocytes transfected with wild-type CD44 cDNA-containing plasmids. The relative expression of each protein was quantified to GAPDH by densitometry and normalized to GAPDH. n = 3 for each group. *p < 0.05 versus control by one-way ANOVA with Bonferroni’s post hoc test. [file 12929_2023_944_MOESM4_ESM.pdf]

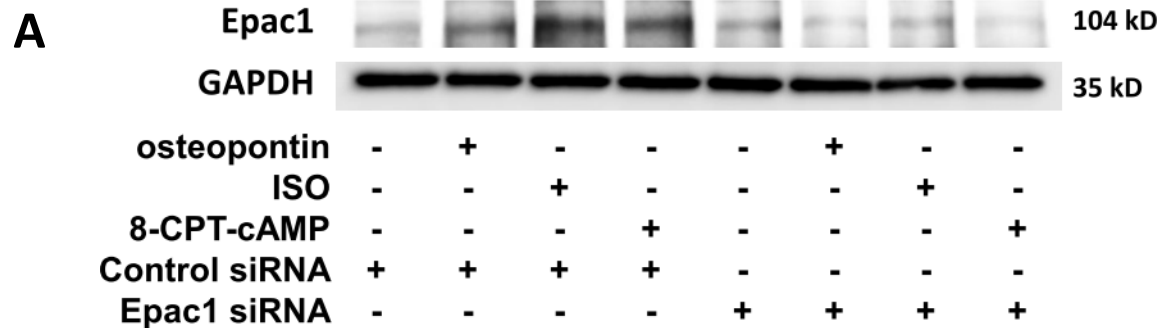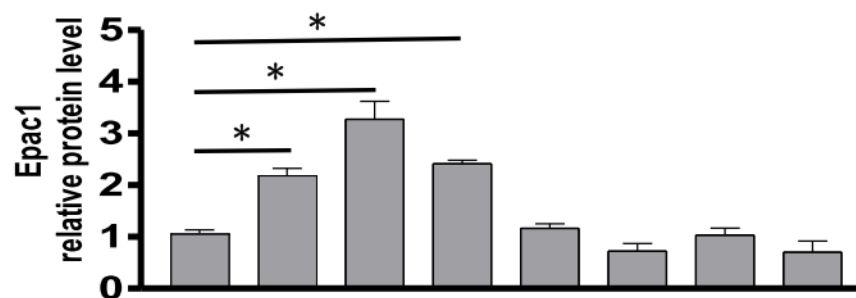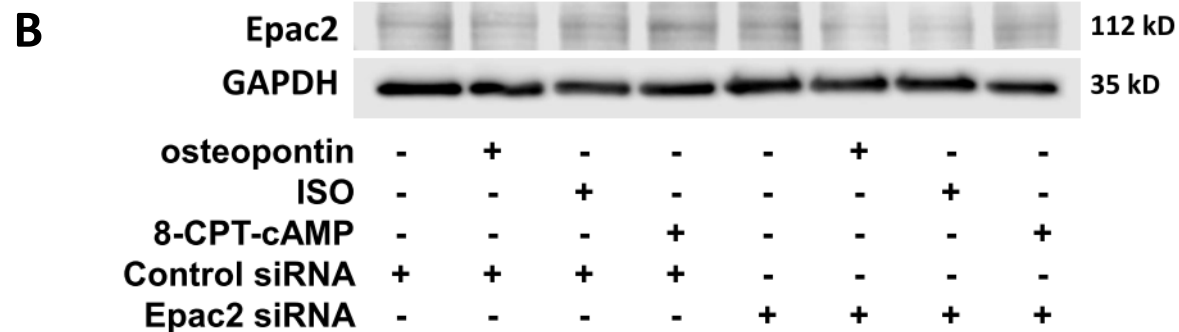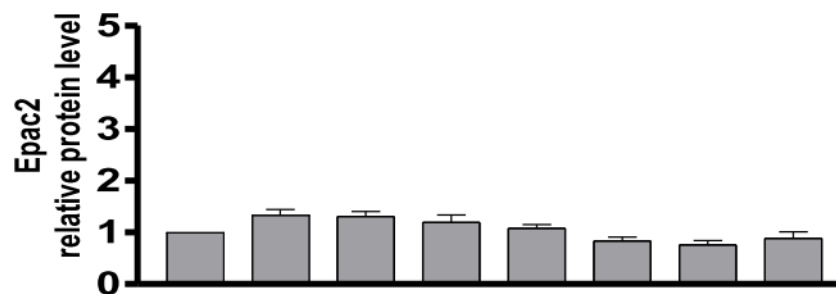

Supplement: Supplementary file 5 — Additional file 5: Figure S4. Effects of osteopontin, β-AR and Epac activation on Epac. Representative examples and mean ± SE analysis western blot for (A) Epac1 and (B) Epac2 in HL-1 myocytes treated with osteopontin, isoproterenol, 8-CPT-cAMP or Epac siRNAs. The relative expression of each protein was quantified to GAPDH by densitometry and normalized to GAPDH. n = 4 for each group. *P < 0.05 versus control by one-way ANOVA with Bonferroni’s post hoc test. ISO = isoproterenol [file 12929_2023_944_MOESM5_ESM.pdf]

**A**

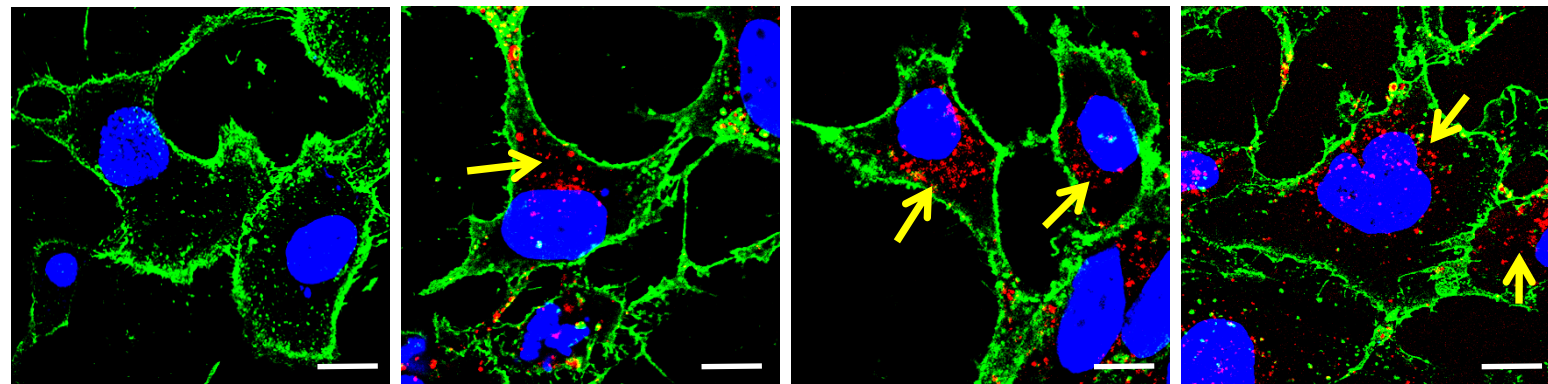

osteopontin

-

+

-

-

ISO

-

-

+

-

8-CPT-cAMP

-

-

-

+

**Control**

**ISO**

**a**

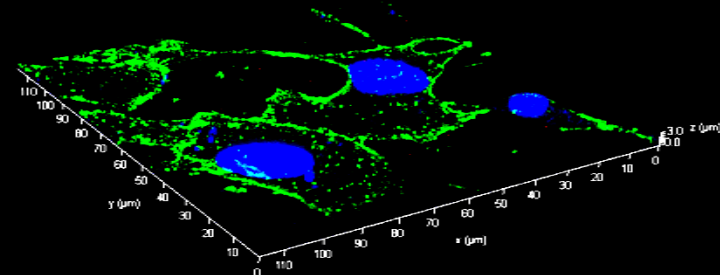

**c**

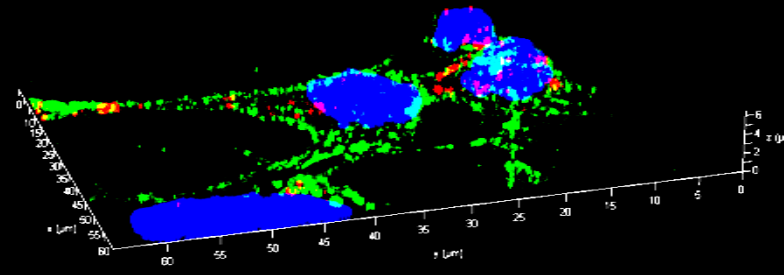

**b**

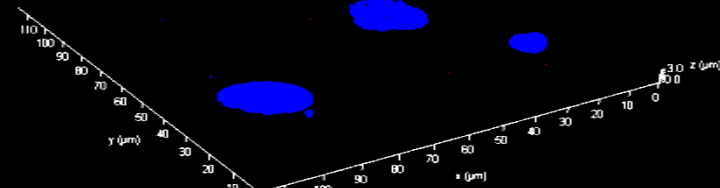

**d**

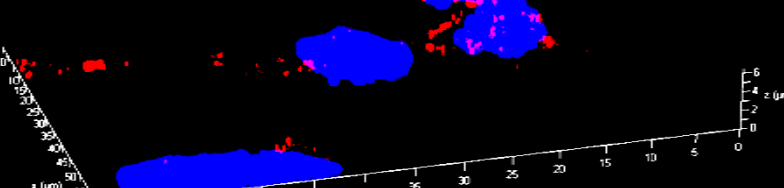

**B**

**Control**

**ISO**

**CD44**

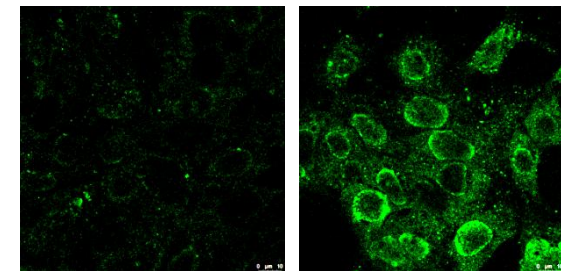

**Epac1**

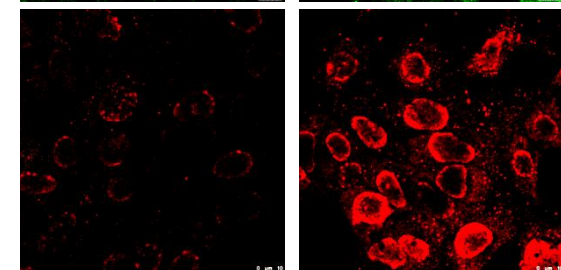

**merge**

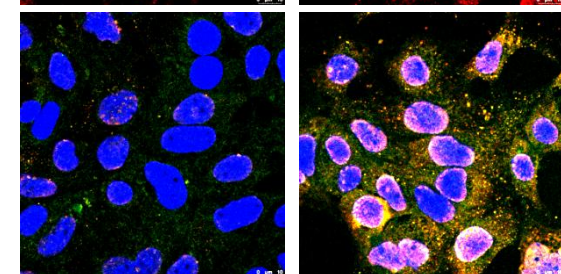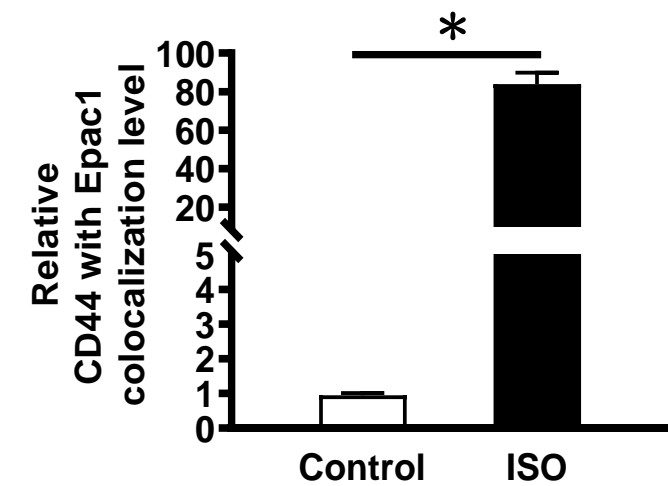

Supplement: Supplementary file 6 — Additional file 6: Figure S5. Proximity ligation assay and co-localization of CD44 and Epac1 in HL-1 myocytes. A. Representative con-focal images of proximity ligation assay with WGA (green color), which was used to mark cell membrane in HL-1 myocytes. The red color (arrows) indicates the association between CD44 and Epac1. Representative examples of z-stacking mode in HL-1 myocytes with (c, d) and without (a, b) treatment with isoproterenol. B. Representative con-focal images of CD44 (upper, green color), Epac1 (middle, red color) and co-localization of both (lower, yellow color) and mean ± SE analysis of co-localization in HL-1 myocytes treated with isoproterenol. The relative fluorescence of co-localization was normalized to control as 1.0. n = 4 images per group. *P < 0.05 versus control by unpaired Student t-test. Bar = 10 μm. ISO = isoproterenol, WGA = wheat germ agglutinin. [file 12929_2023_944_MOESM6_ESM.pdf]

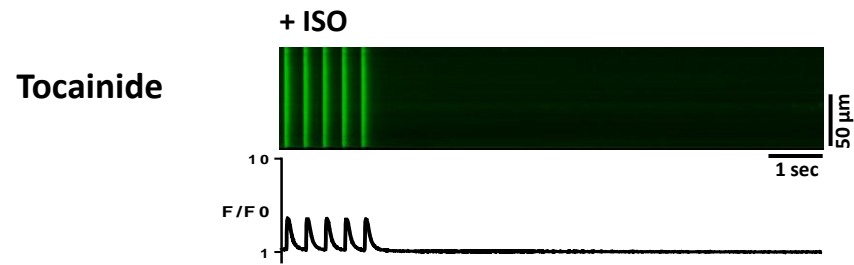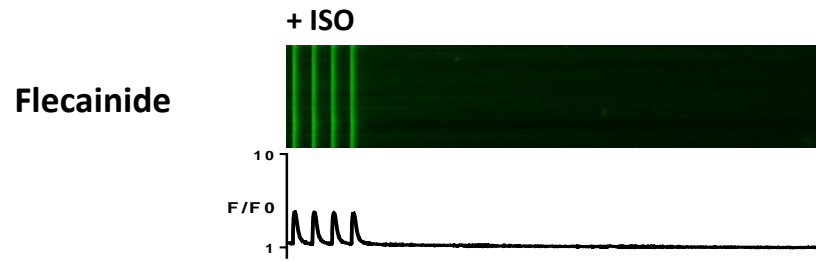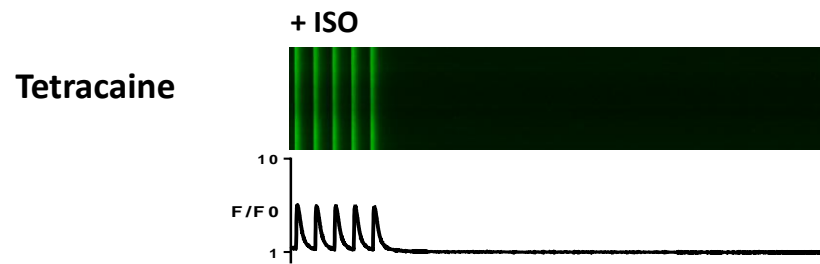

Supplement: Supplementary file 7 — Additional file 7: Figure S6. Representative examples of Ca2+ waves in ventricular myocytes from WT mice pretreated with flecanide (10 μM) for 20 min, tocanide (50 μM) or tetracine (50 μM) for 10 min and after isoproterenol and 3-Hz electrical stimulation. The results were confirmed from 9—18 cells for each group. WT = wild-type mice, ISO = isoproterenol. [file 12929_2023_944_MOESM7_ESM.pdf]

**A**

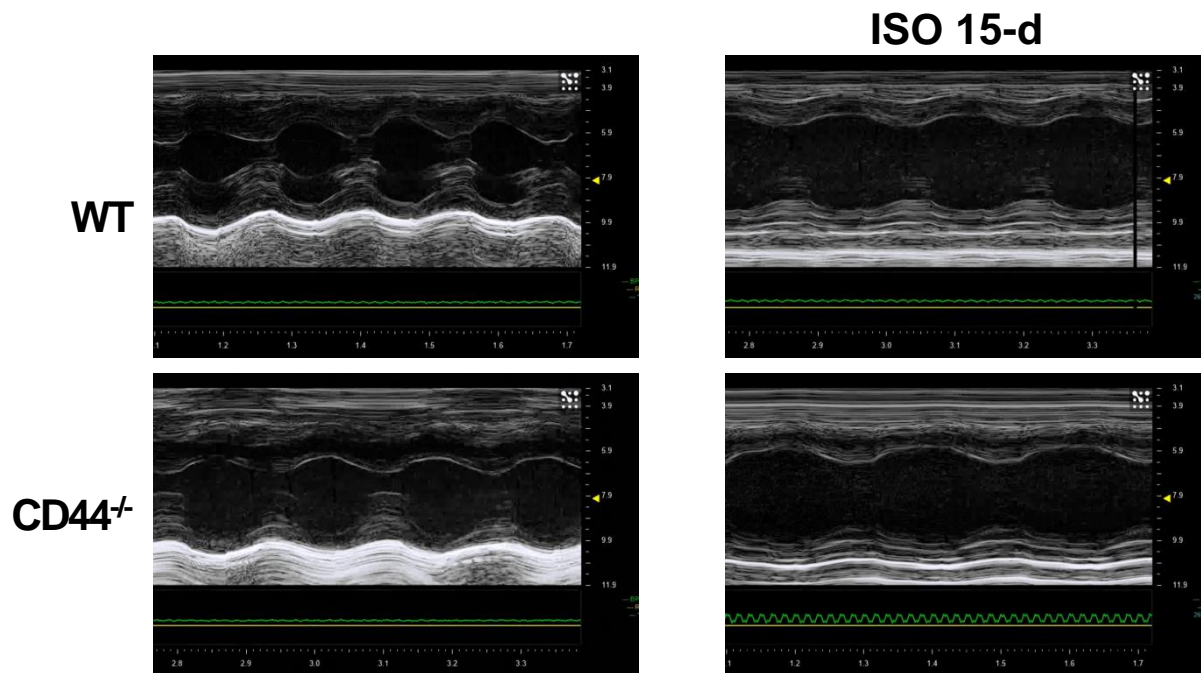

**B**

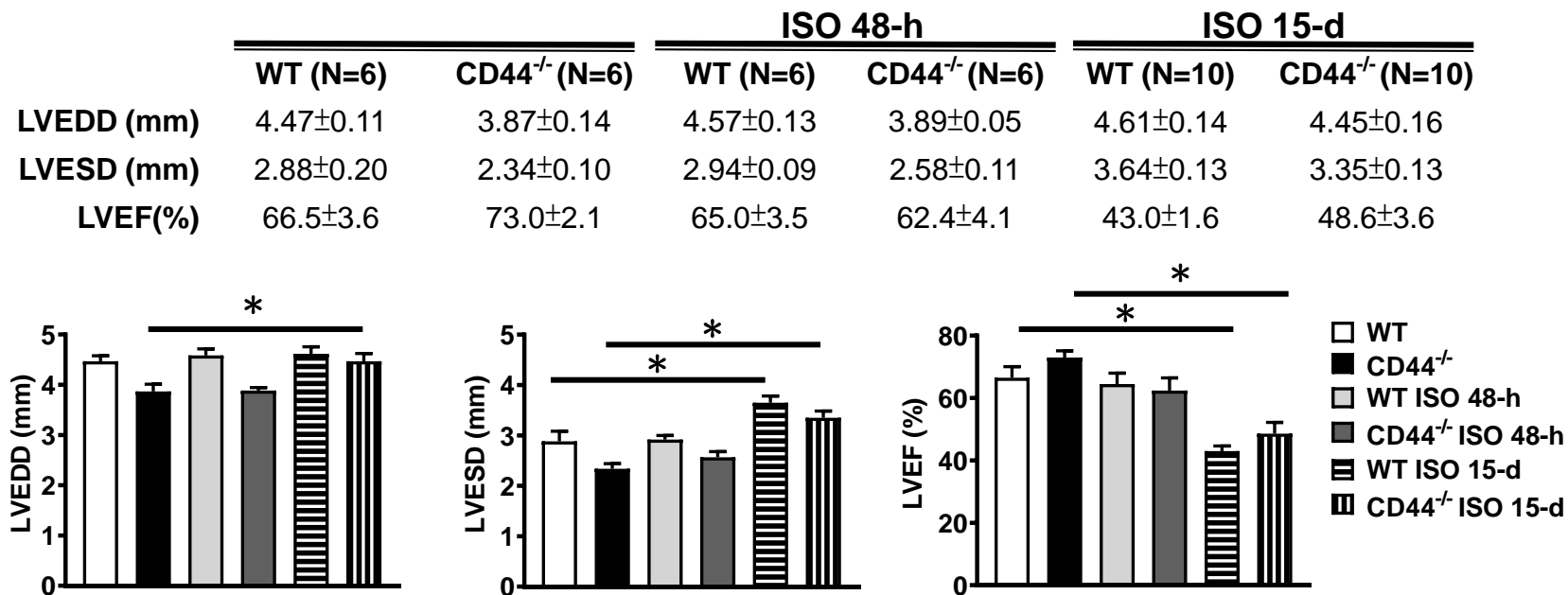

Supplement: Supplementary file 8 — Additional file 8: Figure S7. Examples of M-mode echocardiographic images (A) and mean ± SE analysis for LVEF (B) of the heart from WT and CD44−/− mice at baseline and after treatment with ISO. N = 5 for each group, *p < 0.05 versus control (WT without ISO) by one-way ANOVA with Bonferroni’s post hoc test. WT = wild-type control mice. CD44−/− = CD44 knock-out mice, WT = wild-type control mice, ISO = Isoproterenol at 30 mg/kg/day subcutaneously for 15 days. LVEF = left ventricular ejection fraction. [file 12929_2023_944_MOESM8_ESM.pdf]

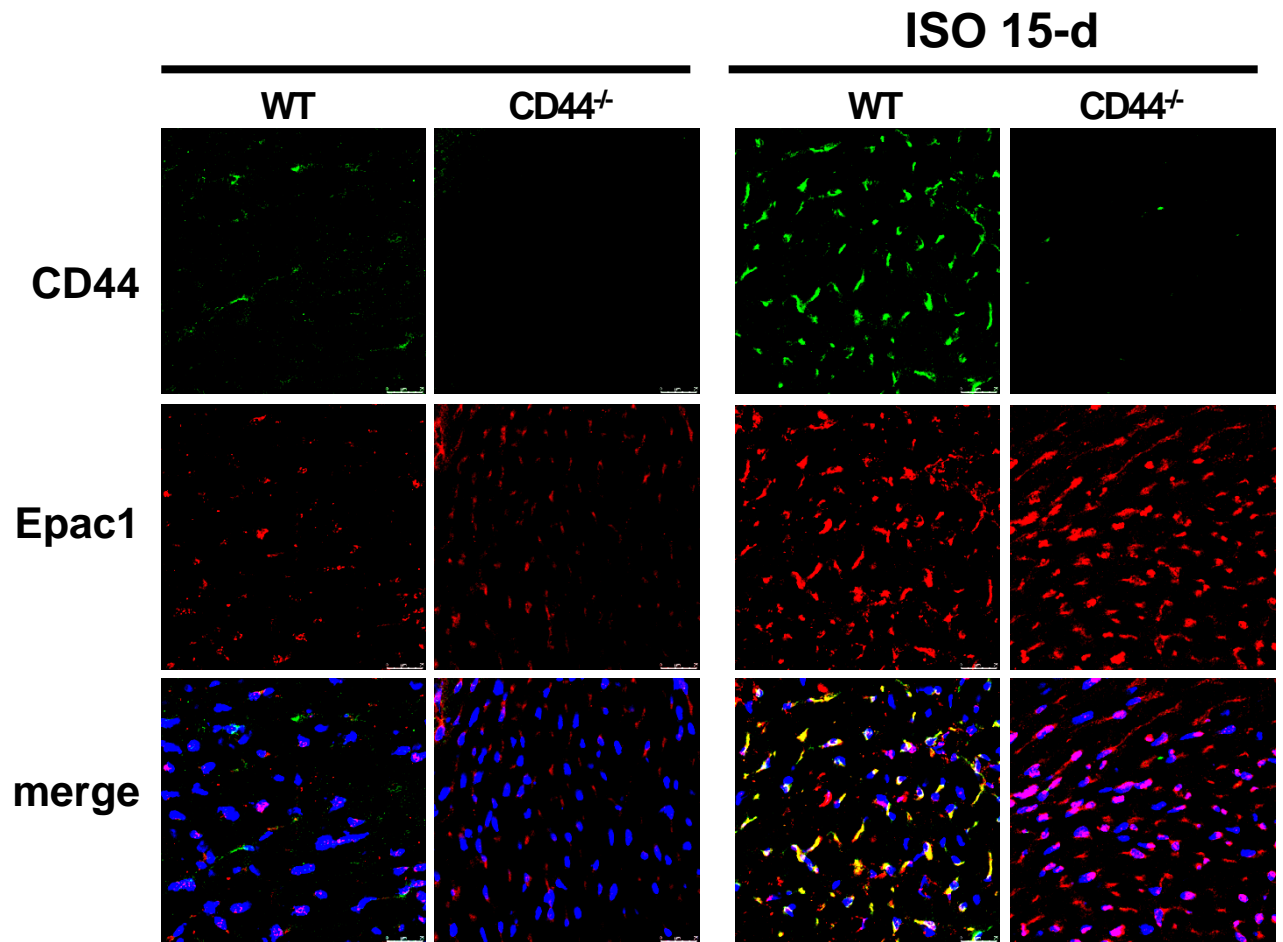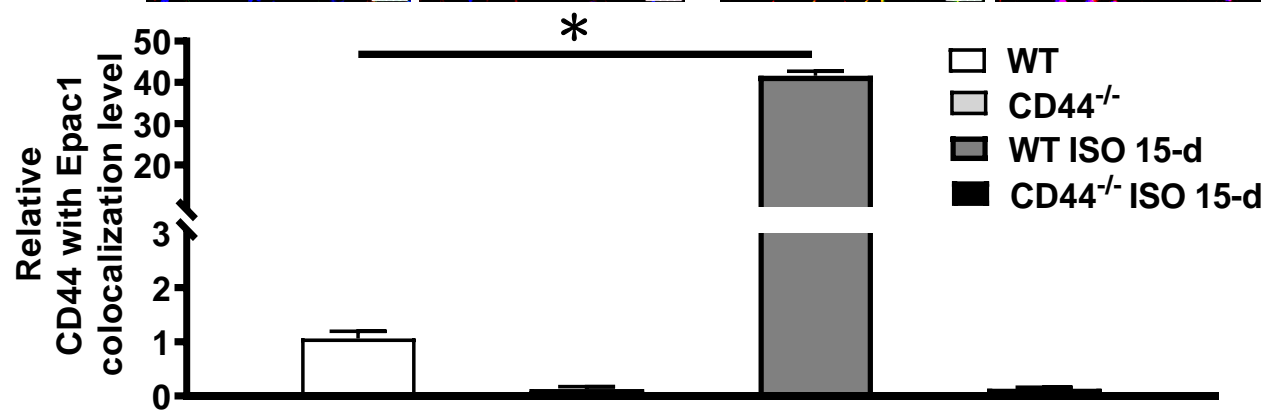

Supplement: Supplementary file 9 — Additional file 9: Figure S8. Co-localization of CD44 and Epac1 in heart failure mice. Representative confocal images of CD44 (upper, green color), Epac1 (middle, red color) and co-localization of both (lower, yellow color) and mean ± SE analysis of co-localization in WT and CD44−/− mice heart at baseline and after treatment with ISO. The relative fluorescence of co-localization was normalized to WT as 1.0. N = 4 per group. *P < 0.05 versus control by one-way ANOVA with Bonferroni’s post hoc test. WT = wild-type control mice, CD44−/− = CD44 knock-out mice, ISO = isoproterenol at 30 mg/kg per day subcutaneously for 15 days, HF = heart failure. [file 12929_2023_944_MOESM9_ESM.pdf]
