# Supplementary material for: CD44 regulates Epac1-mediated β-adrenergic-receptor-induced Ca2+-handling abnormalities: implication in cardiac arrhythmias
Source: J Biomed Sci. 2023 Jul 14;30:55. doi: 10.1186/s12929-023-00944-0 (PMC10347873; doi:10.1186/s12929-023-00944-0)
Supplement: Supplementary file 10 — Additional file 10: Table S1. The electrocardiography parameters in wild-type and CD44−/− mice at baseline and with heart failure [file 12929_2023_944_MOESM10_ESM.doc]

Supplementary Table 1

|  | WT  (N=6) | | | CD44^- / -^  (N=8) | | | WT HF  (N=7) | | | CD44^- / -^ HF  (N=9) | | |
| --- | --- | --- | --- | --- | --- | --- | --- | --- | --- | --- | --- | --- |
| PR interval (ms) | 38.7 | ± | 0.6 | 43.6 | ± | 2.4 | 48.7 | ± | 1.2 | 68.1 | ± | 2.8*^#$^ |
| QRS complex (ms) | 14.7 | ± | 0.4 | 15.2 | ± | 0.6 | 16.7 | ± | 1.1 | 14.2 | ± | 0.7 |
| QT interval (ms) | 44.6 | ± | 2.1 | 50.8 | ± | 1.9 | 52.5 | ± | 1.9 | 49.9 | ± | 1.4 |

| The electrocardiography parameters in wild-type and CD44^-/-^ mice |
| --- |

|  |  |  |  |  |  |  |  |  |  |  |  |  |
| --- | --- | --- | --- | --- | --- | --- | --- | --- | --- | --- | --- | --- |

Values are mean ± standard deviation in all variables; WT, wild-type mice; CD44^-/-^, CD44 knock-out mice; HF, heart failure

* P < 0.05 to WT, # p < 0.05 to CD44-/- and $ p < 0.05 to WT HF by one-way ANOVA.
