# Supplementary material for: CD44 regulates Epac1-mediated β-adrenergic-receptor-induced Ca2+-handling abnormalities: implication in cardiac arrhythmias
Source: J Biomed Sci. 2023 Jul 14;30:55. doi: 10.1186/s12929-023-00944-0 (PMC10347873; doi:10.1186/s12929-023-00944-0)
Supplement: Supplementary file 11 — Additional file 11: Table S2. Characteristics of the patients [file 12929_2023_944_MOESM11_ESM.doc]

| **Supplementary Table 2.** Clinical characteristics of patients | | |
| --- | --- | --- |
| Clinical Variable | Preserved  LV contractility | Compromised  LV contractility |
|  | N = 4 | N = 4 |
| Age, years | 65 ± 16.1 | 63 ± 15.6 |
| Sex (n, %) |  |  |
| Female | 3 (75) | 2 (50) |
| Male | 1 (25) | 2 (50) |
| Parameters for LV diameter | | |
| LVEDD (mm) | 56±7 | 63±11 |
| LVESD (mm) | 39±3 | 50±14 |
| Parameters for LV function | | |
| LVEF, % | 73 ± 3 | 40 ± 8 |
| Hypertension | 2 | 1 |
| Diabetes | 0 | 0 |
| β-blocker | 2 | 1 |
| ARB/ACEi | 0 | 0 |
| Primary MR | 4 | 0 |
| Secondary MR | 0 | 4* |

Values are mean ± standard deviation in all variables except for gender; n, individuals; LV, left ventricle; LVEDD, left ventricular end diastolic diameter; LVESD, left ventricular end systolic diameter; LVEF, left ventricular ejection fraction; ARB, angiotensin II receptor blocker; ACEi, antiogensin converting enzyme inhibitor; MR, mitral regurgitation

* *P* < 0.05 between two groups by Chi-squared test.
